# Investigating the use of pollen DNA metabarcoding to quantify bee foraging and effects of threshold selection

**DOI:** 10.1371/journal.pone.0282715

**Published:** 2023-04-18

**Authors:** Katherine A. Arstingstall, Sandra J. DeBano, Xiaoping Li, David E. Wooster, Mary M. Rowland, Skyler Burrows, Kenneth Frost

**Affiliations:** 1 Department of Fisheries, Wildlife, and Conservation Sciences, Oregon State University, Corvallis, Oregon, United States of America; 2 Hermiston Agricultural Research and Extension Center, Oregon State University, Hermiston, Oregon, United States of America; 3 Department of Botany and Plant Pathology, Oregon State University, Corvallis, Oregon, United States of America; 4 United States Forest Service, Pacific Northwest Research Station, La Grande, Oregon, United States of America; 5 Bee Biology and Systematics Lab, Utah State University, Logan, Utah, United States of America; University of Leipzig Faculty of Life Sciences: Universitat Leipzig Fakultat fur Lebenswissenschaften, GERMANY

## Abstract

DNA metabarcoding of pollen is a useful tool for studying bee foraging ecology. However, several questions about this method remain unresolved, including the extent to which sequence read data is quantitative, which type of sequence count removal threshold to use and how that choice affects our ability to detect rare flower visits, and how sequence artefacts may confound conclusions about bee foraging behavior. To address these questions, we isolated pollen from five plant species and created treatments comprised of pollen from each species alone and combinations of pollen from multiple species that varied in richness and evenness. We used ITS2 and *rbcL* metabarcoding to identify plant species in the samples, compared the proportion of pollen by mass to the proportion of sequencing reads for each plant species in each treatment, and analyzed the sequencing data using both liberal and conservative thresholds. We collected pollen from foraging bees, analyzed metabarcoding data from those samples using each threshold, and compared the differences in the pollinator networks constructed from the data. Regardless of the threshold used, the relationship between the proportion of pollen by mass and sequencing reads was inconsistent, suggesting that the number of sequence reads is a poor proxy for pollen abundance in mixed-species samples. Using a liberal threshold resulted in greater detection of original plant species in mixtures but also detected additional species in mixtures and single-species samples. The conservative threshold reduced the number of additional plant species detected, but several species in mixtures were not detected above the threshold, resulting in false negatives. Pollinator networks produced using the two thresholds differed and illustrated tradeoffs between detection of rare species and estimation of network complexity. Threshold selection can have a major effect on conclusions drawn from studies using metabarcoding of bee pollen to study plant-pollinator interactions.

## Introduction

Bee populations worldwide are currently experiencing significant declines [1, 2], and one way to reverse these declines is to increase the quantity and quality of their habitat by planting flowering species that are important food sources for bees [3]. However, determining which plant species are significant food sources for bees can be challenging. Traditional methods for describing bee-flower interactions (e.g., visual observation, microscopy) are time-consuming, can require specialized expertise, and often lack taxonomic resolution [4–6]. However, the recent use of DNA metabarcoding on bee pollen is potentially a more effective method for identifying food sources for bees than traditional methods, resulting in higher taxonomic resolution and revealing a more detailed record of bee foraging behavior [7–13].

Pollen metabarcoding has become a widely used method that is applicable to many different organisms and areas of research including, but not limited to, allergen monitoring [14], plant biodiversity assessments [15], and biomonitoring of plant pathogens [16]. A number of questions remain about pollen metabarcoding analyses in general and potential limitations of metabarcoding to establish floral resource preferences of bees, specifically. First, the issue of whether sequence read data can be used to quantify the amount of each plant species in a mixed pollen load is unresolved. Over the past decade, researchers have investigated the quantitative abilities of pollen DNA metabarcoding using six different gene regions, three nuclear ribosomal and three plastid, and have yet to come to a consensus (Table 1). Furthermore, studies that use the same barcode markers continue to find contradictory results. For example, Keller et al. [7] found similar relative abundances when comparing ITS2 sequencing reads to microscopic pollen counts, while Richardson et al. [8] found no association.

**Table 1:** Results of studies examining quantitative relationship between attributes of pollen in mixed samples and reads of genetic barcode markers.

Study	Barcode Marker	Support of Quantitative Relationship
Keller et al. [7]	ITS2	Yes—similar relative abundance of reads compared to microscopic counts
Kraaijeveld et al. [17]	*trnL*	Yes—found that correcting the number of sequencing reads by the total number of microscopically counted pollen gave an accurate estimate of the absolute number of each pollen type in a sample
Richardson et al. [8]	ITS2	No—no relationship in relative abundance of reads compared to microscopic counts
Richardson et al. [9]	ITS2, *matK*, and *rbcL*	No—certain species were consistently over- or underrepresented in the molecular results relative to the microscopic results
Pornon et al. [10]	ITS1 and *trnl*	Yes—positive relationship between amount of DNA isolated from each plant species and reads of both markers
Bell et al. [18]	ITS2 and *rbcL*	No—even though significant correlations between sequence reads and proportion of pollen grains for each species, variance explained was low
Richardson et al. [19]	ITS2, *rbcL*, *trnH*, and *trnl*	Yes—statistically significant relationship between microscopy pollen counts and median number of sequence reads for family level
Baksay et al. [20]	ITS1 and *trnL*	Yes—found a significant positive relationship between the number of DNA sequences and the number of pollen grains in mock solutions
Polling et al. [21]	nrITS2 and *trnL*	Yes—found highly significant positive relationships between the relative abundance of sequencing reads and the relative abundance of microscopically obtained pollen concentrations

Sequence read data may not accurately quantify the amount of pollen from each plant species in a mixed pollen load when certain plant species are over- or underrepresented [8, 11, 18]. Misrepresentation might occur for several reasons including variable gene copy number [22, 23], differences in DNA extraction efficiency [24], and primer amplification bias [25, 26]. Brooks et al. [24] found that different combinations of DNA extraction kits and number of polymerase chain reaction (PCR) cycles resulted in dramatically different proportions of sequencing reads per taxa. Additionally, sequence variation at the barcode priming site can affect the amplification efficiency of a plant species, resulting in false negatives [26, 27]. Nevertheless, several studies have found definitive quantitative relationships and proposed that number of sequence reads are a good proxy for the relative abundance of a species in a pollen load [7, 10, 17]. Resolving the issue of whether DNA metabarcoding of bee pollen produces accurate quantitative results will allow us to determine the types of questions that can be answered using this approach and rule out those that cannot.

**Fig 1:**
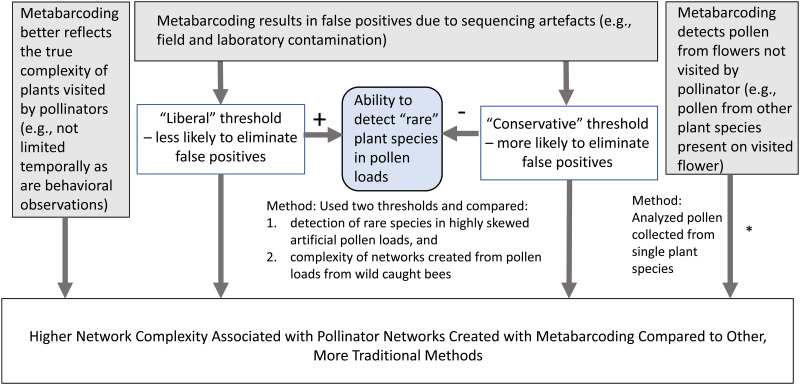
Three general explanations for greater complexity associated with pollinator networks derived from metabarcoding. Three general explanations for the pattern of higher complexity often associated with pollinator networks based on metabarcoding data compared to more traditional methods. Possible explanations are shown in gray boxes. Explanations are not mutually exclusive. The potential impacts of “liberal” and “conservative” thresholds (employed to decrease false positives) on the ability to detect rare species (shown in the blue rounded box) are depicted with signed arrows. “Rare” plant species in the network are those that may be visited by only a few individuals of a particular pollinator species, or may be visited by many species, but at relatively lower rates compared to other plant species. *This process is also influenced by threshold selection, but is not shown to simplify the figure.

Another category of issues that must be addressed relates to how well plant-pollinator networks produced from plant species assignments obtained using metabarcoding data represent pollinator foraging behavior (Fig 1). Several studies have found that plant-pollinator networks based on metabarcoding data are more complex, containing more plant taxa and interactions than networks created using foraging observations [12, 13, 28]. Although this pattern may occur simply because metabarcoding studies provide a more accurate depiction of bee foraging behavior than traditional methods, the tendency of metabarcoding networks to be more complex than those based on foraging observations alone could be influenced by other factors (Fig 1). One potential factor is sequence artefacts associated with field and laboratory processes (e.g., contamination) (Fig 1). Contamination that occurs in the field and laboratory is often addressed by using negative controls at each stage of the metabarcoding process (DNA isolation, PCR, plating), which can be used in various ways to quantify the number of sequencing reads in a sample that can be attributed to contamination alone. The number of sequencing reads found in negative controls can be used to create a sequence count removal threshold, and any taxonomic assignment whose read count falls below this threshold can be considered contamination or other “background noise” and removed from further analysis [18]. However, there is currently no standardized protocol for creating these thresholds in pollen metabarcoding studies, despite the fact that different threshold protocols can potentially yield different interpretations of the same results.

One general approach is to remove entire plant taxa from the dataset if the total number of reads for the taxa is less than a threshold. For example, Richardson et al. [19] discarded “taxonomic groups represented by <0.01% of the data” after first removing any taxonomic groups that were assigned using only one of the four barcode markers in the study. Similarly, Pornon et al. [10] eliminated plant species with less than 1,000 sequencing reads. In contrast, another general approach is to compare the number of reads found in each individual taxonomic assignment to a threshold. Bell et al. [18] took this approach by using the maximum number of sequencing reads in their negative controls as their threshold, and setting any taxonomic assignment in a sample with fewer reads than this threshold to zero. Macgregor et al. [29] also used the maximum number of reads in their controls to create their sequence count removal threshold and set any individual assignment falling below it to zero. Because one approach removes entire taxonomic groups while the other removes specific plant-pollinator interactions, different results and interpretations of pollinator behavior may result. Although multiple studies have used similar versions of the same techniques, threshold choice appears to be somewhat arbitrary. Identifying a standardized threshold protocol would help eliminate these problems and allow for comparison of pollinator foraging behavior (e.g., plant-pollinator networks) across multiple pollen metabarcoding studies.

An additional consideration in selecting a contamination threshold approach is that its choice also influences the likelihood of eliminating rare interactions occurring at low frequencies [22] (Fig 1). One way to examine the potential trade-off associated with threshold selection and rare species detection is to sequence laboratory mixtures with plant species present in amounts small enough to represent a rare plant-pollinator interaction and compare each threshold’s ability to detect these species. This approach could help determine whether rare interactions can be detected using DNA metabarcoding of pollen or if the number of sequencing reads produced by the pollen collected during these rare interactions might fall below the sequence count removal threshold.

Another way that metabarcoding could potentially overestimate pollinator network complexity is by detecting pollen dispersed by wind and other insects [30–32]. A flower could have pollen present from several other plant species before a pollinator visits it (Fig 1). Thus, a pollinator could inadvertently pick up pollen from multiple plant species in one flower visit. Despite this possibility, no studies have isolated pollen collected from flowering plants in natural settings and sequenced their pollen to determine if additional plant species were detected and how that detection is affected by the type of contamination threshold used.

Here, we collected pollen by hand from five plant species to create single-species pollen samples and five mixtures of known concentrations, varying in species richness (3–5 species) and evenness (uniform to highly skewed). We used DNA metabarcoding of the ITS2 gene and *rbcL* region, separately, to identify the plant species in the samples, and analyzed the sequencing data using both a liberal and a conservative sequence count removal threshold. We also collected pollen from foraging bees and used metabarcoding data from those samples to construct plant-pollinator networks to compare consequences of using the two thresholds. The specific objectives of this study were to determine whether 1) the proportions of plant species in a pollen mixture based on mass correspond with proportions of plant species in a pollen mixture based on number of sequencing reads, 2) the two types of thresholds yield different results with regard to detecting rare plant-bee interactions and portraying pollinator network complexity, and 3) metabarcoding detects additional plant species in single-species pollen samples, and if so, how that is influenced by threshold selection.

## Materials and methods

Plant and invertebrate materials were collected on public lands managed jointly by the U.S. Forest Service Wallowa-Whitman National Forest and Pacific Northwest Research Station, with their permission. Such collections do not require a field permit or special use authorization.

### Flower collection

Flowers were collected in July 2017 from the United States Forest Service (USFS) Starkey Experimental Forest and Range (Starkey) and from Hermiston, both located in eastern Oregon. Slender cinquefoil (*Potentilla gracilis*), Oregon checkerbloom (*Sidalcea oregana*), and mountain goldenbanner (*Thermopsis montana*) flowers were collected at Starkey (45.2332°N, 118.5511°W). Starkey is a long-term research site in Union County (elevation 1,130–1,500 m) that was established in 1940 [33]. Sampling took place at sites along Meadow Creek, a major tributary of the Upper Grande Ronde River that flows through Starkey. Hairy vetch (*Vicia villosa*) and Scotch thistle (*Onopordum acanthium*) flowers were collected in Hermiston (45.8169°N, 119.2846°W). Flowers from each plant species were collected by hand and stored in separate plastic bags. Each bag contained flowers sampled from multiple plants of the same species. Flowers were stored at -20°C until further processing.

### Isolating pollen from flowers

All tools used during pollen isolation were placed in 10% bleach for at least one min prior to use and in between each bout of pollen isolation to reduce contamination. Stamens were removed from each flower using forceps and placed in a glass vial filled with sterilized water. All stamens from each plant species were placed in the same vial. Vials were shaken vigorously for one min to detach pollen from anthers. The solution was poured through a fine mesh sieve into a vacuum filtration system. The pollen was collected on a 5-μm mixed cellulose ester filter. The filters were then placed in a drying oven at 65°C for approximately two hours. Once dry, filters were placed in individual 50 mL centrifuge tubes, and 10 mL of acetone was added to each tube. The centrifuge tubes were vortexed until the filters were completely dissolved, and the solution was mixed thoroughly. The tubes were then centrifuged for 3 min at 2,000 rpm (448 x g), and the supernatant was discarded. Then, 1 mL of acetic acid was added to each tube, and the pellet was re-suspended. The solution was transferred to a 1.5 mL microcentrifuge tube. The solution was centrifuged for 30 s at 12,500 rpm (14,403 x g), and the supernatant was discarded. Two washes were performed with 1 mL of sterilized water for 30 s at 12,500 rpm (14,403 x g). This was followed by two washes with 1 mL of ethanol for 30 s at 12,500 rpm (14,403 x g) and 3 min at 13,500 rpm (15,555 x g). The supernatant was discarded, and the pollen pellet was dried for 30 min. The mass of the dried pollen pellet was calculated as ((mass of pollen pellet + microcentrifuge tube)—mass of microcentrifuge tube) and recorded.

### Preparation of single-species samples and mixtures

Single-species samples were created for each of the five plant species (three replicates each) by transferring ~1.5 mg of the stock pollen to a 1.5 mL screw cap microcentrifuge tube using a scoopula and a balance scale. All tools were placed in 10% bleach for at least one min prior to use and between different plant species. Next, a ~10 mg stock was created for each of the five pollen mixtures representing a range of species richness (3–5 species) and evenness (uniform to highly skewed). Mixture 1 was a slightly skewed mixture with pollen from three different plant species, mixture 2 was a slightly skewed mixture with pollen from four different plant species, mixture 3 was a uniform mixture with pollen from all five plant species, and mixtures 4 and 5 were highly skewed mixtures with pollen from all five plant species. See table in S1 Table for total mass and proportions of each mixture stock. Mixture stocks were vortexed for 30 s to ensure even concentration of each species. We transferred ~2.5 mg of pollen from each mixture stock to a 1.5 mL screw cap microcentrifuge tube as described above (three replicates of each mixture). Mass of samples and rank abundance of plant species within the samples were determined based on the amount of pollen isolated from each plant species.

### Bee sampling

Bees of varying taxa were sampled during peak foraging hours (0900–1800) from Starkey on June 25, 2017. Each bee was either collected directly into an individual glass vial or caught with an insect net and then transferred into a vial. Each bee was given a unique identification number so that it could be associated with the metabarcoding data obtained from its pollen load. Bees were pinned, sexed and identified to species using the methods described in Kuhlman and Burrows [34].

### Isolating pollen from bees

Pollen grains were isolated from each bee’s body by either shaking vigorously in a vial filled with sterilized water or scraping pollen loads from the corbicula directly into a vial for small/medium sized bees and large bees, respectively. Small/medium bees were removed from the vials, and the pollen solution was transferred to a 50 mL centrifuge tube. Each 50 mL tube was centrifuged for 4 min at 2,000 rpm (448 x g), and the supernatant was discarded. We added 1 mL of water to each 50 mL centrifuge tube to resuspend the pollen pellet. The solution was transferred to a 1.5 mL microcentrifuge tube that was weighed beforehand. The 1.5 mL centrifuge tubes were centrifuged for 3 min at 13,500 rpm (15,555 x g), and the supernatant was discarded. The pollen pellets were dried for 1 hour in an Eppendorf vacufuge. The mass of the dried pollen pellet was calculated as described above and recorded. Bee specimens and pollen pellets were stored at -20°C until further processing.

### DNA extraction and PCR

DNA were extracted from pollen samples using the Macherey-Nagel Nucleospin Food kit (Macherey-Nagel, Bethlehem, Pennsylvania, USA), following the “isolation of genomic DNA from honey or pollen” supplementary protocol. The samples were homogenized in a Mini-BeadBeater-24 (Biospec Products, Bartlesville, Oklahoma, USA). Negative controls (i.e., sterilized water in place of pollen) were included with each round of DNA extraction. For library preparation, we used internal transcribed spacer (ITS2) primers ITS S2F and ITS4R [35] and universal ribulose-1,5-biphosphate carboxylase/oxygenase large subunit (*rbcL*) primers, rbcL2 [36] and rbcLaR [37]. The ITS2 and rbcL barcodes were amplified via a two-step PCR protocol. In the first step, Illumina overhang adapter sequences were added to each primer [38]. Each PCR reaction contained 10 μL of 5X Green GoTaq^®^ Reaction Buffer, 0.3 μL of 10 mM dNTPs, 1 μL of each primer, 0.2 μL of GoTaq^®^ DNA Polymerase, 35.5 μL of water, and 2 μL of the template DNA with a total volume of 50 μL per reaction. The PCR began with an initial heat activation period of 3 min at 95°C, followed by 35 cycles of 30 s at 95°C, 30 s at 55°C, and 1 min at 72°C. A final extension of 10 min at 72°C was included after the last cycle, and the samples were held at 10°C until further processing. Negative controls consisting of water instead of template DNA were included in each round of PCR. A 5 μL sample of each PCR product was electrophoresed in a 2% agarose gel, stained with GelRed^™^ (Biotium Inc, Fremont, CA), and visualized under UV light to confirm the presence of the appropriately sized amplicons. PCR products were purified using the Promega Wizard SV Gel and PCR Clean-Up System and quantified using a Nanodrop 2000 Spectrophotometer (Thermo Scientific, Waltham, MA). Samples were diluted with sterilized water in 96-well plates then shipped to the Center for Genome Research and Biocomputing at Oregon State University for index PCR and sequencing (standard Illumina MiSeq paired-end 300 bp run).

### Bioinformatics

#### Read quality filtering and denoising

A total of 4,530,819 and 3,579,511 raw, paired-end reads were retrieved across 112 samples for ITS2 and *rbcL* respectively. The open-source Quantitative Insights Into Microbial Ecology (QIIME2 version 2020.11 and 2021.2, [39]) was used as our pipeline environment for sequence processing and analysis. The QIIME2 DADA2 plugin [40] was used to filter read quality and denoise reads (mixture ITS: --p-trunc-len-f 254, --p-trunc-len-r 199, mixture *rbcL*: --p-trunc-len-f 300, --p-trunc-len-r 269, 13-pollen samples ITS: --p-trunc-len-f 254, --p-trunc-len-r 202, mixture *rbcL*: --p-trunc-len-f 300, --p-trunc-len-r 269). Noisy reads and reads with a median sequencing quality score below 30 were omitted. Paired-end reads were joined to create contigs, and the duplicated sequences and sequences with chimera were removed. This resulted in a feature table containing optimized sequences and amplicon sequence variants (ASV), which was used for downstream analyses (e.g., taxonomic classification). After filtering and denoising, 2,637,043 (58%) and 2,639,554 (74%) paired-end reads were retained for ITS2 and *rbcL*, respectively.

#### Reference database construction

We used a list of plant species known to occur at Starkey, developed over multiple years by botanists, and generated a list of vascular plant species, including cultivated specimens, known to occur in Umatilla County via the Consortium of Pacific Northwest Herbaria (https://www.pnwherbaria.org). After removing sedges, rushes, and grasses, the lists were comprised of 492 and 1,435 plant species known to be present at Starkey and in Umatilla County respectively. A master plant list was developed that included the 1,607 unique species occurring at both sampling locations. After removal of subspecies and cultivars, the master list was comprised of 1,277 unique species. Reference sequences of the ITS2 and *rbcL* region for the plants included in this list were then retrieved from the National Center for Biotechnology Information (NCBI) database using a python package: NCBI-Companion (available at https://github.com/lixiaopi1985/NCBI_Companion). The downloaded reference sequences were further cleaned to remove duplicates. The ITS2 database contained 13,306 sequences with a median length of 607 bp and the *rbcL* database contained 10,766 sequences with a median length of 607 bp. Respectively, the ITS2 and *rbcL* databases covered 80.4% and 77.1% of the total species on the master list.

#### Training classifier and taxonomy classification

The Naïve Bayes algorithm provided by the QIIME2 feature-classifier plugin [41] was used to train a classifier for taxonomic assignment based on the ITS2 and *rbcL* databases. The default settings were applied (i.e., kmer length = 7, confidence threshold = 0.7) with high accuracy and low recall. The classify-sklearn tool of the QIIME2 feature-classifier plugin [41] was used to import the reference sequences and then assign taxonomy to each ASV using the default settings.

#### Sequence count removal thresholds

We used two types of sequence count removal thresholds to filter the sequencing data: a liberal threshold and a conservative threshold. For the liberal threshold, we removed any taxa whose total number of DNA sequences was less than 0.01% of the total number of DNA sequences for all taxa combined, similar to the methods used by Pornon et al. [10] and Richardson et al. [19]. We categorize this approach as “liberal” because only plant species that are globally rare in the dataset are removed (not interactions that may be rare in a particular pollen load).

For the conservative threshold, we found the average and the standard deviation of the number of sequencing reads in the negative control samples. We added 1.645 standard deviations to the average and used this number as our sequence count removal threshold, accounting for 95% of possible “background noise” detected in the pollen samples [42]. We set the sequence count to zero for any taxonomic assignment whose read count fell below the threshold, similar to the methods used by Bell et al. [18] and Macgregor et al. [29]. We categorize this approach as “conservative” because taxonomic assignments that are rare in each pollen load are removed.

### Comparing proportion of pollen mass and sequencing reads

For each mixture sample, we filtered the sequence reads using both the liberal and conservative threshold. Then, we created stacked bar plots from each data set comparing the proportion of pollen by mass to the proportion of ITS2 and *rbcL* sequencing reads for each plant species within the mixture. Stacked bar plots were created using the ggplot2 package in R [43, 44].

For each of the five plant species included in the mixture samples, we created scatter plots with proportion of pollen by mass on the x-axis and proportion of sequence reads on the y-axis. These scatter plots allowed us to visually compare the relationship between mass of pollen and number of sequence reads to a one-to-one relationship for each plant species, which is what we would expect to see if number of sequence reads were a good proxy for amount of pollen in a sample. Scatter plots were created for both the liberal and conservative threshold data sets in Excel (Version 2211).

### Plant-pollinator network comparison

We filtered the ITS2 and *rbcL* metabarcoding data from the bee pollen loads using both liberal and conservative thresholds and created plant-pollinator networks from both data sets using the bipartite package in R [43, 45]. The total number of plant species and average number of plant species per pollen load were compared among networks.

## Results

### ITS2 metabarcoding

Using plant species assignments obtained from ITS2 metabarcoding, three of the five plant species included in the laboratory-prepared mixtures (*O*. *acanthium*, *S*. *oregana*, and *V*. *villosa*) were correctly identified to the species level. *P*. *gracilis* was assigned only at the genus level, and *T*. *montana* was assigned as *Thermopsis rhombifolia*. These results were consistent for all replicates in each of the five mixtures.

Using the liberal threshold, nine additional plant taxa that were not used to create the laboratory-prepared mixtures were detected above the sequence count removal threshold in the single-species samples (S2 Table). Trace amounts of *O*. *acanthium* and *T*. *montana* (misidentified as *T*. *rhombifolia*) were detected in all single-species samples (S2 Table). All “rare” plant species in mixture 5 were detected above the liberal threshold (S3 Table).

Using the conservative threshold, some plant species in the mixtures were not consistently detected above the sequence count removal threshold, resulting in false negatives (S3 Table). *O*. *acanthium* and *T*.*montana* (misidentified as *T*. *rhombifolia*) were always detected above the sequence count removal threshold (S3 Table). *S*. *oregana*, however, was not detected above the sequence count removal threshold in mixtures 2–5 (S3 Table). *P*. *gracilis* and *V*. *villosa* were not detected above the sequence count removal threshold in mixture 5, and the number of sequencing reads for *P*. *gracilis* fell below the threshold for one replicate of mixture 3 (S3 Table). Only one additional plant species that was not used to create the laboratory-prepared mixture was detected above the sequence count removal threshold in the *P*. *gracilis* single-species samples (S2 Table).

### *rbcL* metabarcoding

Only one of the five plant species included in the laboratory-prepared mixtures (*V*. *villosa*) was correctly identified at the species level using plant species assignments obtained from *rbcL* metabarcoding. *O*. *acanthium* was assigned as *Hieracium* sp., *S*. *oregana* was assigned as *Malva neglecta*, and *P*. *gracilis* and *T*. *montana* were assigned only at the genus level. These results were consistent across all replicates in each mixture.

Using the liberal threshold, 19 additional plant taxa that were not used to create the laboratory-prepared mixtures were detected above the sequence count removal threshold in the single-species samples (S2 Table). Trace amounts of *Thermopsis* sp. were detected in all single-species samples (S2 Table). All “rare” plant species in mixture 5 were detected above the threshold (S3 Table).

Using the conservative threshold, *S*. *oregana* (misidentified as *M*. *neglecta*) was not detected above the sequence count removal threshold in mixtures 2–5, and the number of sequence reads for *P*. *gracilis* fell below the threshold for replicate 3 of mixture 5 (S3 Table). *O*. *acanthium* (misidentified as *Hieracium* sp.), *Thermopsis* sp., and *V*. *villosa* were always detected above the sequence count removal threshold (S3 Table). Four additional plant species that were not used to create the laboratory-prepared mixtures were detected above the sequence count removal threshold in the single-species samples (S2 Table), and trace amounts of *P*. *gracilis* were detected above the sequence count removal threshold in the *S*. *oregana* (misidentified as *M*. *neglecta*) single-species samples (S2 Table).

### Comparing proportion of pollen mass and sequencing reads

**Fig 2:**
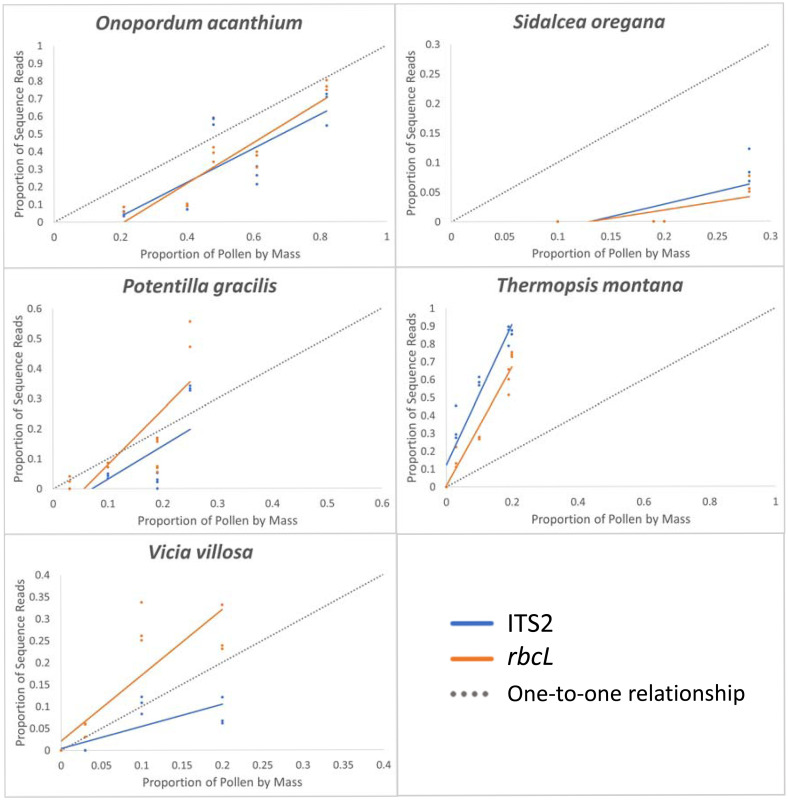
Proportion of pollen by mass in mixtures vs. proportion of sequence reads using the conservative threshold. Gray dotted line represents expected one-to-one relationship. For ITS2, *Potentilla gracilis* = *Potentilla* sp.; *Thermopsis montana* = *Thermopsis rhombifolia*. For *rbcL*, *Onopordum acanthium* = *Hieracium* sp.; *Sidalcea oregana* = *Malva neglecta*; *Potentilla gracilis* = *Potentilla* sp.; *Thermopsis montana* = *Thermopsis* sp. See scatterplots in S1 Fig for liberal threshold results.

**Fig 3:**
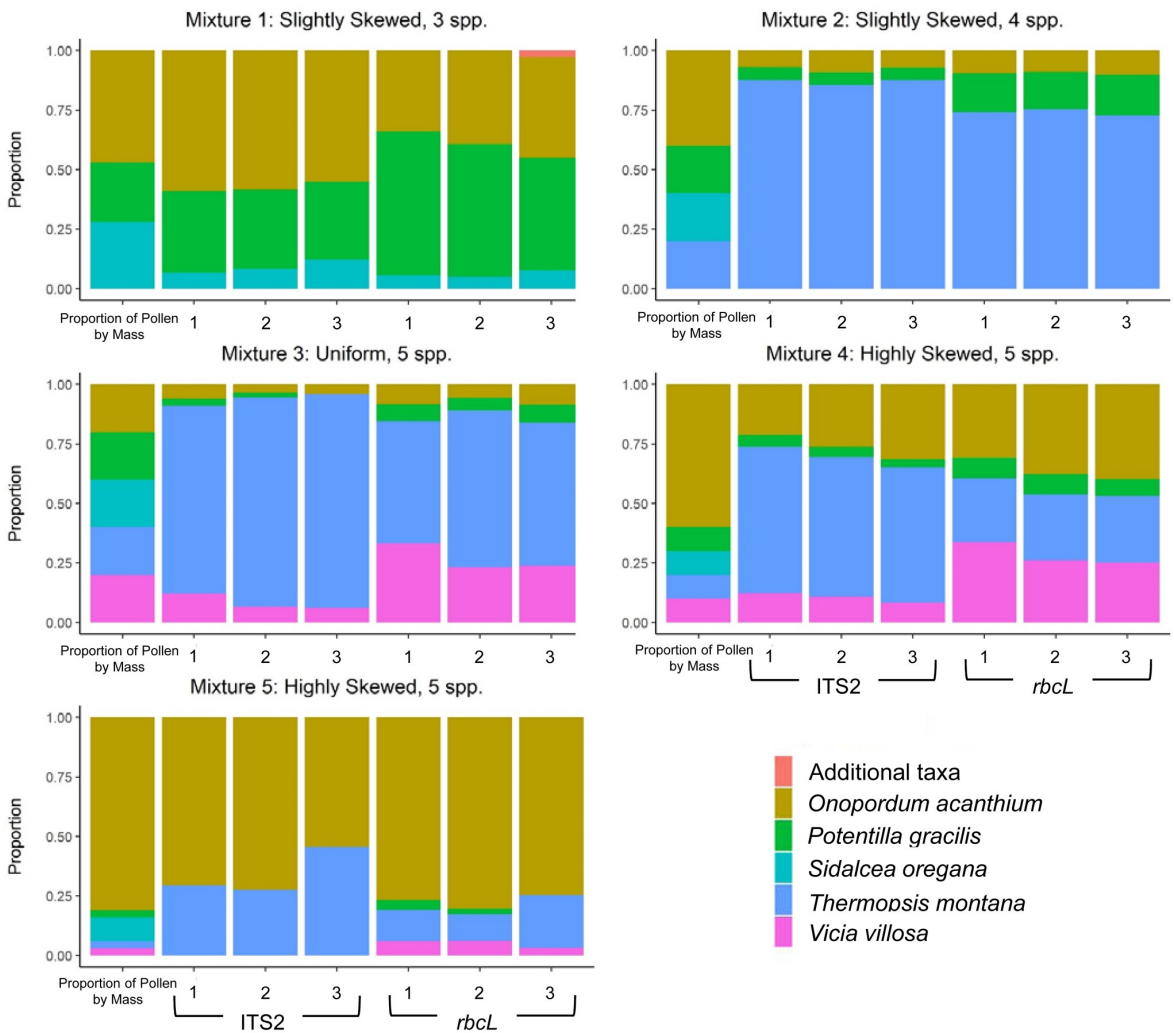
Actual proportion of pollen by mass vs. proportion of sequencing reads using the conservative threshold. The first column of each graph shows the actual proportion of pollen by mass for each plant species in the mixture and the following six columns show the proportion of sequencing reads produced for each species for each of three replicates per mixture obtained with DNA metabarcoding for ITS2 and *rbcL* regions, respectively. For ITS2, *Potentilla gracilis* = *Potentilla* sp.; *Thermopsis montana* = *Thermopsis rhombifolia*. For *rbcL*, *Onopordum acanthium* = *Hieracium* sp.; *Sidalcea oregana* = *Malva neglecta*; *Potentilla gracilis* = *Potentilla* sp.; *Thermopsis montana* = *Thermopsis* sp. See graphs in S2 Fig for liberal threshold results.

Although there was a positive relationship between the proportion of pollen by mass and the proportion of sequencing reads for each species across all mixtures, regardless of the type of threshold used or the region examined, the proportion of pollen of some species was consistently over- or underrepresented by sequence reads (Fig 2). *T*. *montana* was consistently overrepresented by the number of sequencing reads for both regions and *S*. *oregana* was consistently underrepresented by the number of sequencing reads in mixtures for both regions (Fig 2). The consequences of certain species being over- and underrepresented resulted in a poor correspondence between the proportion of pollen by weight and sequence reads for many of the mixtures (Fig 3), with even the ranked abundance of read proportions for species differing from the rank abundance of weight proportions.

### Plant-pollinator network comparison

**Fig 4:**
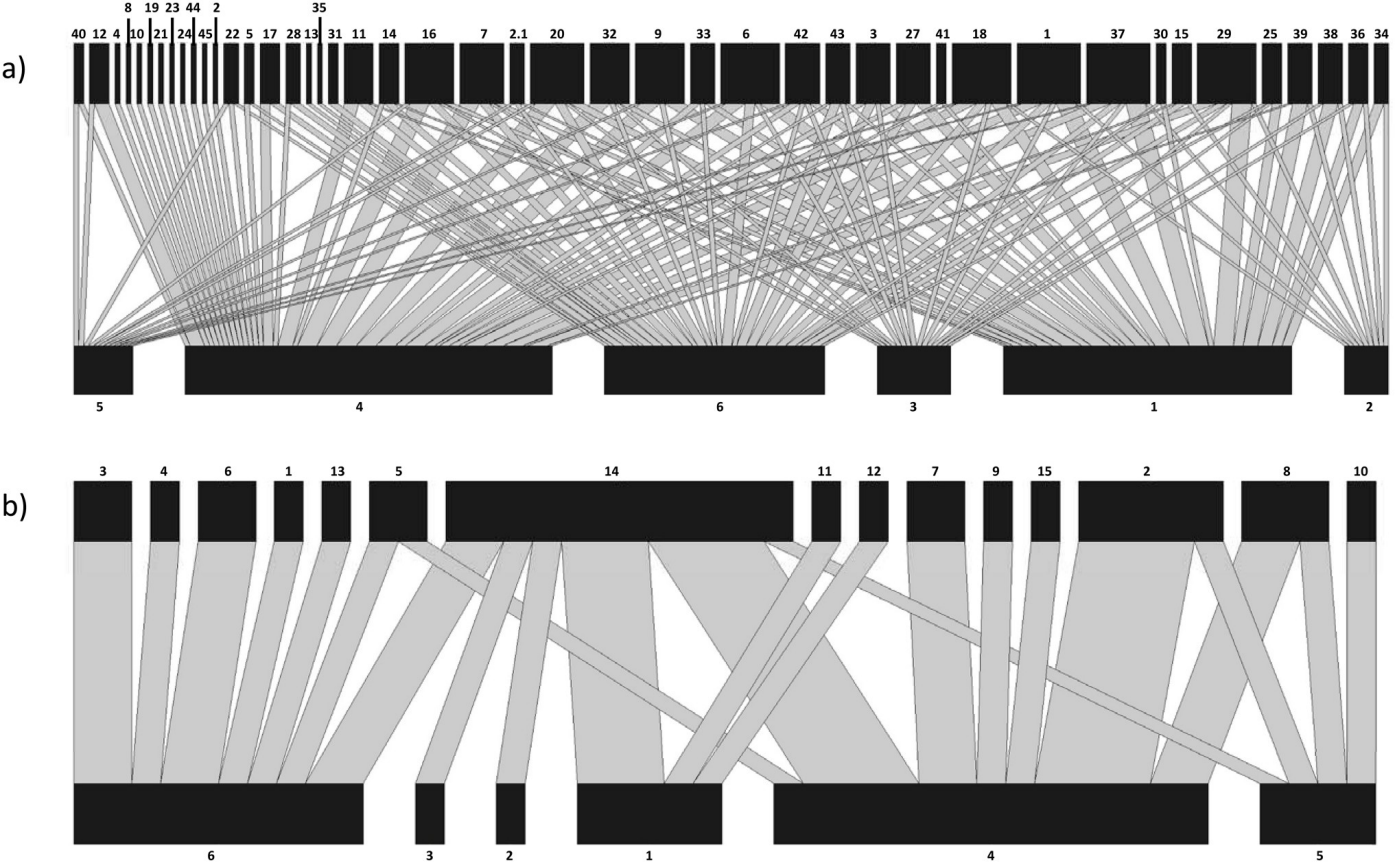
Plant-pollinator networks based on ITS2 and *rbcL* metabarcoding data using liberal and conservative thresholds. (a) Metabarcoding data filtered using the liberal threshold. (b) Metabarcoding data filtered using the conservative threshold. In each network, the top row represents plant taxa and the bottom row represents bee species. The thickness of the lines represents the frequency of the interactions. A complete list of bee species can be found in the table in S5 Table. A complete list of plant species in Fig 4a and 4b can be found in the tables in S6 and S7 Tables, respectively.

We used pollen collected from 13 individual bees representing six species to construct plant-pollinator networks (Fig 4 and S5 Table). When using the liberal threshold to filter ITS2 and *rbcL* metabarcoding results, 44 plant taxa were detected and individual pollen loads contained an average of 16.5 taxa (Fig 4a and S6 Table). When using the conservative threshold, 15 plant taxa were detected and individual pollen loads contained an average of 2.7 taxa (Fig 4b and S7 Table).

## Discussion

The results of our study further document that the number of sequence reads cannot be reliably used to estimate the proportion of pollen from different plant species in a pollen mixture. Some species were consistently overrepresented while others were consistently underrepresented (Figs 2 and 3). One overrepresented species was *T*. *montana*, which had proportions of reads that were on average 4–6 times greater than the proportion of pollen by mass for ITS2 and *rbcL* results regardless of the threshold type. In contrast to *T*. *montana*, the proportion of sequencing reads produced for *S*. *oregana* was on average 33–39 times lower than the proportion of pollen by mass when using the liberal threshold for ITS2 and *rbcL*. Moreover, this species was not even detected above the sequence count removal threshold in 80% of the mixture samples when using the conservative threshold. These deviations from a one-to-one relationship occurred for both barcode regions we examined, even though several studies have suggested that chloroplast barcode markers may be more useful than nuclear ribosomal markers for interpreting metabarcoding results quantitatively [8, 18].

There are several reasons why the number of sequencing reads produced for a species may not be proportional to the mass of pollen including differences in DNA extraction efficiency, amplification bias [26, 27], and differences in marker copy number [23, 46]. Although we used the same DNA extraction kit for all samples, extraction bias can relate to the samples themselves. We were able to extract about eight times the amount of DNA, on average, from *T*. *montana* samples compared to *S*. *oregana* samples of approximately the same size (S8 Table), which corresponds to the over-representation of *T*. *montana* and under-representation of *S*. *oregana*. Bias can also be related to the success of amplification resulting from the barcode’s universal primers. Depending on the combination of barcode marker and species, sequence variation at the priming site can prevent efficient annealing of the primer for that species, resulting in false negatives. Furthermore, amplification efficiency can differ from species to species depending on the composition of their variant of the barcode sequence, resulting in proportions of sequence reads that differ from the actual proportion of the species in the sample [12]. Finally, marker copy number could have contributed to over- and under-representation of *T*. *montana* and *S*. *oregana*, respectively. ITS2 is a multi-copy gene region, with high variation in copy number among different plant species [47], and marker copy number bias for chloroplasts is likely to be high when working with pollen because of the various modes of chloroplast inheritance for different plant lineages [48].

Regardless of the mechanism responsible for the over- or under-representation of sequence reads for particular plant species relative to the actual proportion of pollen in a mixed load, our findings show that the issue presents a serious obstacle to using sequence read data quantitatively to assess mixed species samples. When researchers use DNA metabarcoding to determine which plant species are in a pollen sample, the reference library to which they compare the DNA sequences in their samples may contain hundreds or even thousands of plant species, depending on the study. Attempts to derive quantitative information from pollen samples would require predetermination of which plant species may be over- or underrepresented. Since quantitative biases of certain species are dependent on the other species present in the sample [10, 18], every combination of species would need to be tested prior to analysis, a task that would be practically impossible. Several researchers have proposed the use of correction factors to derive quantitative information from DNA metabarcoding sequence reads [8, 17, 49]. However, the proportions of plant species in a mixed pollen sample are affected by negative correlation bias and thus cannot be examined independently [50]. Although sequencing reads produced by pollen metabarcoding cannot be used to quantify the amount of a plant species in a mixed pollen sample using current methods, sequencing technologies are rapidly advancing and this type of quantification may be possible in the near future using other methods like genome-skimming and whole-genome shotgun sequencing [51, 52].

The results of this study also illustrate the importance of developing reference libraries that correspond as closely as possible to the sampling areas of interest to increase the likelihood of correctly identifying pollen to the species level. Although the five plant species used to create the laboratory-prepared mixtures were consistently detected above the liberal threshold using both ITS2 and *rbcL* barcode markers, several species were misidentified (S9 Table). These misidentifications were likely due to the lack of strong interspecific genetic divergence at the ITS2 and *rbcL* barcoding sites. This explanation is more likely than misidentified reference sequences in the database because genus and family level identifications were consistently correct for ITS2 and *rbcL* DNA sequences, respectively. In our study, plant species used to create the pollen mixtures were collected from two locations in eastern Oregon. One location (Starkey) has a well-documented plant list specific to the area. In contrast, the plant list generated for the other site (Hermiston) was based on a much larger geographic area (i.e., the 1,435 plant species known to occur in Umatilla County). In our study, it is likely that a large number of plants included on the county list do not occur in the particular area that we sampled. Using regional lists instead of site-specific lists to create reference databases increases the likelihood of erroneous identifications when using metabarcoding [13]. Using a narrower list of plant species known to occur in our sampling location rather than a county-wide list of plant species when creating our reference database would have likely led to more accurate plant species assignments.

Another major issue that our study addressed was the degree to which methods used to decrease erroneous conclusions about bee foraging and pollinator network complexity influence rare species detection (Fig 1). When using DNA metabarcoding to identify plant species in a pollen mixture, a certain amount of contamination is inevitable. Contamination can occur at any stage of the DNA metabarcoding process, so precautions are taken in the field and laboratory (e.g., using clean nets to catch each bee, bleaching workspaces and tools). Additional steps are then taken to remove potential sequencing artefacts (e.g., negative controls, sequence count removal thresholds). However, methods that are designed to eliminate sequencing artefacts are also likely to decrease the detection of rare plant species that occur in lower quantities in a pollen load (e.g., from rare flower visits by bees), as these species may fall below the sequence count removal threshold and subsequently be removed from analysis.

Our results suggest that the detection of rare bee-flower interactions depends on the type of threshold used. Our most highly skewed mixture contained 0.075 mg (3%) of pollen from each of *P*. *gracilis*, *T*. *montana*, and *V*. *villosa*, which represent rare bee-flower interactions in a pollen load. When using our liberal threshold, which removes any plant taxa whose total number of sequencing reads falls below 0.01% of total reads, all “rare” plant species were detected above the threshold. However, when using the conservative threshold, *S*. *oregana* was not detected above the sequence count removal threshold in any of the highly skewed mixture replicates (mixtures 4 and 5). Furthermore, *P*. *gracilis* and *V*. *villosa* were not detected above the sequence count removal threshold in any of the mixture 5 replicates when using the ITS2 barcode marker and the conservative threshold. Therefore, it appears that we can consistently detect “rare” bee-flower visits when using the liberal threshold, but not when using the conservative threshold. However, using a conservative threshold may not always present a serious limitation when studying plant-pollinator interactions because rare bee-flower visits may not be ecologically significant. Certain steps can be taken to reduce stochastic PCR effects such as performing library-prep PCRs in replicate and pooling them [7, 15, 25]. This may have helped us detect more rare species when using the conservative threshold, and we recommend that future studies perform this step, if feasible.

While many precautions can be taken in the laboratory to avoid and control for contamination issues, contamination is more difficult to control in the field. Wind and other insects can move pollen around the environment before sampling occurs. Pollen from other plant species could be present on a flower before a bee visits, resulting in the bee picking up pollen from multiple plant species in one visit. If this were the case, we would expect to see multiple plant species that were not part of our artificial mixtures in our single-species samples. When using the liberal threshold, nine and 19 additional plant species were detected in the single-species samples when using ITS2 and *rbcL* barcode markers, respectively. When using the conservative threshold, only one and four additional plant species were detected in the single-species samples based on ITS2 and *rbcL* sequencing results, respectively. It is important to note that we pooled pollen from flowers from several different individual plants when creating our single-species pollen samples, which likely contributed to the number of additional plant species detected in these samples. However, regardless of the number of additional species detected, the dataset filtered with the liberal threshold consistently included a greater number of additional species than the dataset filtered with the conservative threshold, suggesting that the use of a liberal threshold may overestimate resources use by bees.

We see the consequences of this in the plant-pollinator networks created using each threshold type (Fig 4). The network created using data filtered by the liberal threshold had three times as many plant species than the network created using data filtered by the conservative threshold. In addition, bees, on average, were found to be carrying six times the number of plant species in their pollen loads when using the liberal threshold compared to the conservative one. The ecological interpretations of these results are very different: in Fig 4a, the bee species appear to be exhibiting generalist foraging behavior, whereas in Fig 4b, they appear to be much more selective in their choice of foraging resources. Furthermore, these interpretations lead to different management actions regarding the creation and maintenance of pollinator habitat. Overestimation of network complexity may be a common problem in studies that use DNA metabarcoding to examine bee foraging behavior, and it is important to be aware of the trade-off between using a liberal or conservative sequence count removal threshold with regard to the detection of “rare” plant species and false positives.

## Conclusions

In this study, certain plant species were consistently over- and underrepresented by the number of ITS2 and *rbcL* sequence reads produced for the laboratory-created pollen mixtures and rank abundance of sequencing reads did not correspond to rank abundance of pollen mass. This suggests that DNA metabarcoding of the ITS2 and *rbcL* gene region cannot be used to estimate the abundance of a plant species in a mixed pollen sample and rank abundance of sequencing reads should not be used as a proxy for rank abundance of pollen. Furthermore, although one could argue that the plastid barcode marker used in this study appeared to perform slightly better than the nuclear ribosomal marker, its performance was not improved enough to justify using plastid barcode markers to interpret pollen metabarcoding data quantitatively. Although some misidentification occurred, we were able to consistently detect plant species present in amounts representing rare bee-flower visits when using a liberal threshold, but several additional plant taxa that were not used to create the laboratory-prepared mixtures were also detected in the single-species samples. When using a conservative threshold, some false negatives occurred, but we detected significantly fewer additional plant taxa in the single-species samples. Based on the information presented here, a conservative threshold seems to be most appropriate for the study of most plant-pollinator interactions. When describing these interactions, we suggest that it would generally be better to not capture a few rare flower visits that may not be ecologically relevant than to include potential field contamination and other background noise that could significantly skew results. However, we realize that certain research questions may require the use of a liberal sequence count removal threshold (e.g., detection of a specific compound collected in small amounts). We encourage future researchers using pollen metabarcoding to study plant-pollinator interactions to critically examine their threshold choice to determine whether it is appropriate for the questions bring addressed. Finally, we recommend that all pollen metabarcoding studies clearly report the methods used to create and use sequence count removal thresholds so that results can be compared across multiple studies and used to provide accurate and consistent data to inform management decisions regarding pollinator habitat.

## Supporting information

S1 Fig Proportion of pollen by mass in mixtures vs. proportion of sequence reads for using the liberal threshold. Gray dotted line represents expected one-to-one relationship. For ITS2, *Potentilla gracilis* = *Potentilla* sp.; *Thermopsis montana* = *Thermopsis rhombifolia*. For *rbcL*, *Onopordum acanthium* = *Hieracium* sp.; *Sidalcea oregana* = *Malva neglecta*; *Potentilla gracilis* = *Potentilla* sp.; *Thermopsis montana* = *Thermopsis* sp.
(TIF)

S2 Fig Actual proportion of pollen by mass vs. proportion of sequencing reads using the liberal threshold. The first column of each graph shows the actual proportion of pollen by mass for each plant species in the mixture and the following six columns show the proportion of sequencing reads produced for each species for each of three replicates per mixture obtained with DNA metabarcoding for ITS2 and *rbcL* regions, respectively. For ITS2, *Potentilla gracilis* = *Potentilla* sp.; *Thermopsis montana* = *Thermopsis rhombifolia*. For *rbcL*, *Onopordum acanthium* = *Hieracium* sp.; *Sidalcea oregana* = *Malva neglecta*; *Potentilla gracilis* = *Potentilla* sp.; *Thermopsis montana* = *Thermopsis* sp.
(TIF)

S1 Table Mass (proportion) of each plant species in the mixture stocks used to create the mixture replicates. Mass is measured in milligrams.
(DOCX)

S2 Table Number (proportion) of ITS2 and *rbcL* sequencing reads for each plant species in single-species samples (three replicates/sample) using liberal and conservative thresholds. See table in S4 Table for a list of additional taxa identified in pollen samples. Additional taxa are defined as plant taxa detected in the samples that were not used to create the laboratory-prepared pollen mixtures. See table in S9 Table for taxonomic assignments using ITS2 and *rbcL* metabarcoding.
(DOCX)

S3 Table Proportion by mass and number (proportion) of ITS2 and *rbcL* sequencing reads for each plant species in laboratory-prepared mixtures (three replicates/mixture) using liberal and conservative thresholds. Shaded boxes represent instances in which the conservative threshold failed to detect species in mixture, resulting in false negatives. See table in S4 Table for a list of additional taxa identified in mixture samples. Additional taxa are defined as plant taxa detected in the samples that were not used to create the laboratory-prepared pollen mixtures. See table in S9 Table for taxonomic assignments using ITS2 and *rbcL* metabarcoding.
(DOCX)

S4 Table Plant taxa that were not used to create the laboratory-prepared mixtures but were detected in mixtures and single-species samples. Laboratory-prepared mixtures were created from the same pollen stocks used to create the single-species samples. “M” denotes a plant taxon that was detected in a mixture sample, “S” denotes a plant taxon that was detected in a single-species sample, and “MS” denotes a plant taxon that was detected in both a mixture sample and a single-species sample.
(DOCX)

S5 Table Complete list of bee species included in Fig 4. (DOCX)

S6 Table Complete list of plant taxa included in Fig 4a. (DOCX)

S7 Table Complete list of plant taxa included in Fig 4b. (DOCX)

S8 Table Mass and DNA yield for each pollen sample. (DOCX)

S9 Table Plant taxonomic assignments from ITS2 and *rbcL* metabarcoding. (DOCX)

S10 Table Plant species and corresponding number of sequencing reads found in each of the negative controls. Extraction, PCR and Plate refers to negative controls that were collected during DNA isolation, PCR, and plating respectively.
(DOCX)

S1 File Complete lists of plant species detected in each individual bee pollen load. Worksheets of plant taxonomic assignments retained after using a liberal or conservative sequence count removal threshold for each individual bee pollen load.
(XLSX)
