# Supplementary figures and images for: Investigating the use of pollen DNA metabarcoding to quantify bee foraging and effects of threshold selection
Source: PLoS One. 2023 Apr 18;18(4):e0282715. doi: 10.1371/journal.pone.0282715 (PMC10112814; doi:10.1371/journal.pone.0282715)

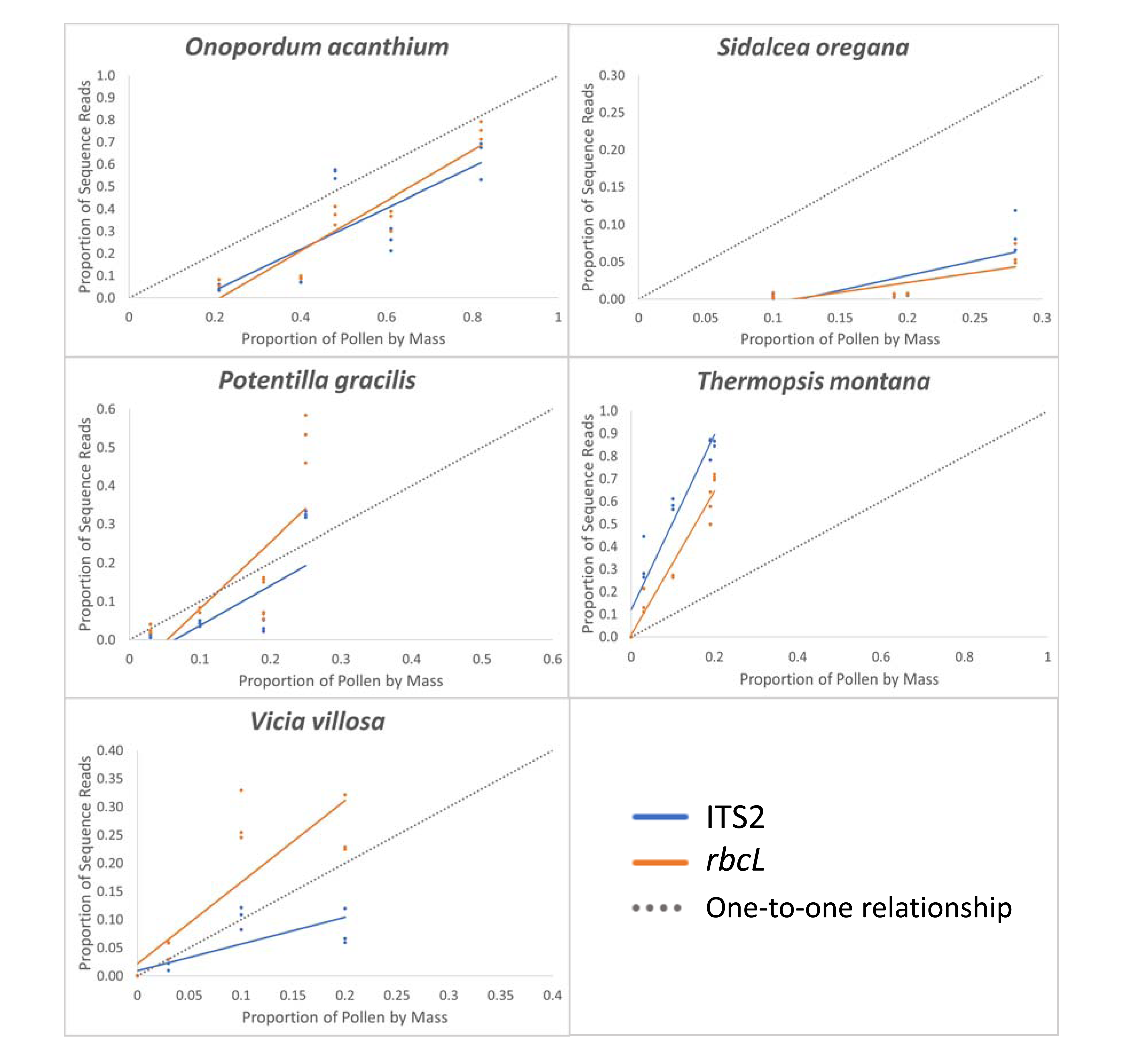

Supplement: S1 Fig — Gray dotted line represents expected one-to-one relationship. For ITS2, Potentilla gracilis = Potentilla sp.; Thermopsis montana = Thermopsis rhombifolia. For rbcL, Onopordum acanthium = Hieracium sp.; Sidalcea oregana = Malva neglecta; Potentilla gracilis = Potentilla sp.; Thermopsis montana = Thermopsis sp. (TIF) [file pone.0282715.s001.tif]

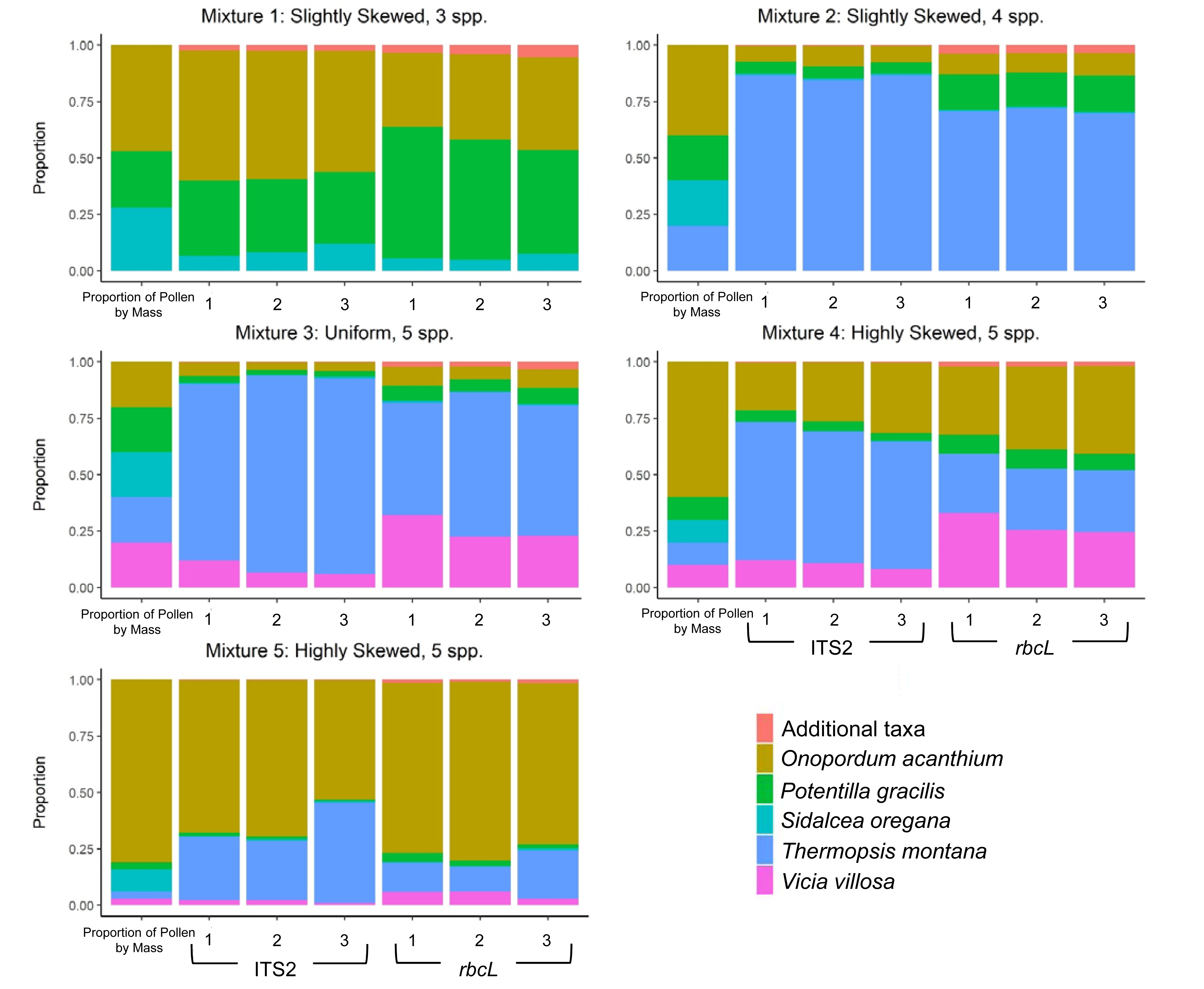

Supplement: S2 Fig — The first column of each graph shows the actual proportion of pollen by mass for each plant species in the mixture and the following six columns show the proportion of sequencing reads produced for each species for each of three replicates per mixture obtained with DNA metabarcoding for ITS2 and rbcL regions, respectively. For ITS2, Potentilla gracilis = Potentilla sp.; Thermopsis montana = Thermopsis rhombifolia. For rbcL, Onopordum acanthium = Hieracium sp.; Sidalcea oregana = Malva neglecta; Potentilla gracilis = Potentilla sp.; Thermopsis montana = Thermopsis sp. (TIF) [file pone.0282715.s002.tif]
