# Supplementary material for: Investigating the use of pollen DNA metabarcoding to quantify bee foraging and effects of threshold selection
Source: PLoS One. 2023 Apr 18;18(4):e0282715. doi: 10.1371/journal.pone.0282715 (PMC10112814; doi:10.1371/journal.pone.0282715)
Supplement: S1 Table — Mass is measured in milligrams. (DOCX) [file pone.0282715.s003.docx]

**S1 Table. Mass (proportion) of each plant species in the mixture stocks used to create the mixture replicates.**

|  | ***Onopordum acanthium*** | ***Sidalcea oregana*** | ***Potentilla gracilis*** | ***Thermopsis montana*** | ***Vicia villosa*** | **Total** |
| --- | --- | --- | --- | --- | --- | --- |
| **Mixture 1** | 5.0 (0.476) | 2.9 (0.276) | 2.6 (0.248) | NA | NA | 10.5 (1.0) |
| **Mixture 2** | 4.1 (0.398) | 2.1 (0.204) | 2.0 (0.194) | 2.1 (0.204) | NA | 10.3 (1.0) |
| **Mixture 3** | 2.2 (0.214) | 2.0 (0.194) | 2.0 (0.194) | 2.0 (0.194) | 2.1 (0.204) | 10.3 (1.0) |
| **Mixture 4** | 6.2 (0.608) | 1.0 (0.098) | 1.0 (0.098) | 1.0 (0.098) | 1.0 (0.098) | 10.2 (1.0) |
| **Mixture 5** | 8.3 (0.815) | 1.0 (0.098) | 0.3 (0.029) | 0.3 (0.029) | 0.3 (0.029) | 10.2 (1.0) |

Mass is measured in milligrams.
