# Supplementary material for: Investigating the use of pollen DNA metabarcoding to quantify bee foraging and effects of threshold selection
Source: PLoS One. 2023 Apr 18;18(4):e0282715. doi: 10.1371/journal.pone.0282715 (PMC10112814; doi:10.1371/journal.pone.0282715)
Supplement: S2 Table — See table in S4 Table for a list of additional taxa identified in pollen samples. Additional taxa are defined as plant taxa detected in the samples that were not used to create the laboratory-prepared pollen mixtures. See table in S9 Table for taxonomic assignments using ITS2 and rbcL metabarcoding. (DOCX) [file pone.0282715.s004.docx]

**S2 Table. Number (proportion) of ITS2 and *rbcL* sequencing reads for each plant species detected in single-species samples (three replicates/sample) using liberal and conservative thresholds.**

| **Single-Species Sample** | **Replicate** | **Species Composition** | **Number (proportion) of ITS2 sequence reads** | | **Number (proportion) of rbcL sequence reads** | |
| --- | --- | --- | --- | --- | --- | --- |
|  |  |  | Liberal threshold | Conservative threshold | Liberal threshold | Conservative threshold |
| *Onopordum acanthium* | 1 | *Onopordum acanthium* | 40,235  (0.9991) | 40,235  (1.0) | 69,475  (0.9759) | 69,475  (1.0) |
|  |  | *Sidalcea oregana* | 0 | 0 | 207  (0.0029) | 0 |
|  |  | *Potentilla gracilis* | 0 | 0 | 0 | 0 |
|  |  | *Thermopsis montana* | 19  (0.0005) | 0 | 37  (0.0005) | 0 |
|  |  | *Vicia villosa* | 0 | 0 | 7  (0.0001) | 0 |
|  |  | Additional taxa | 17  (0.0004) | 0 | 1,465  (0.0206) | 0 |
|  | 2 | *Onopordum acanthium* | 35,618  (0.9985) | 35,618  (1.0) | 56,651  (0.9607) | 56,651  (1.0) |
|  |  | *Sidalcea oregana* | 0 | 0 | 712  (0.0121) | 0 |
|  |  | *Potentilla gracilis* | 0 | 0 | 10  (0.0002) | 0 |
|  |  | *Thermopsis montana* | 14  (0.0004) | 0 | 41  (0.0007) | 0 |
|  |  | *Vicia villosa* | 0 | 0 | 225  (0.0038) | 0 |
|  |  | Additional taxa | 39  (0.0011) | 0 | 1,330  (0.0226) | 0 |
|  | 3 | *Onopordum acanthium* | 37,791  (0.9979) | 37,791  (1.0) | 68,643  (0.9605) | 68,643  (1.0) |
|  |  | *Sidalcea oregana* | 0 | 0 | 759  (0.0106) | 0 |
|  |  | *Potentilla gracilis* | 0 | 0 | 0 | 0 |
|  |  | *Thermopsis montana* | 21  (0.0006) | 0 | 27  (0.0004) | 0 |
|  |  | *Vicia villosa* | 13  (0.0003) | 0 | 200  (0.0028) | 0 |
|  |  | Additional taxa | 46  (0.0012) | 0 | 1,840  (0.0257) | 0 |
| *Sidalcea oregana* | 1 | *Onopordum acanthium* | 15  (0.0006) | 0 | 122  (0.0020) | 0 |
|  |  | *Sidalcea oregana* | 25,100  (0.9693) | 25,100  (1.0) | 45,809  (0.7326) | 45,809  (0.7679) |
|  |  | *Potentilla gracilis* | 406  (0.0157) | 0 | 5,325  (0.0852) | 5,352  (0.0893) |
|  |  | *Thermopsis montana* | 38  (0.0015) | 0 | 15  (0.0002) | 0 |
|  |  | *Vicia villosa* | 0 | 0 | 20  (0.0003) | 0 |
|  |  | Additional taxa | 335  (0.0129) | 0 | 11,240  (0.1798) | 8,519  (0.1428) |
|  | 2 | *Onopordum acanthium* | 47  (0.0022) | 0 | 248  (0.0055) | 0 |
|  |  | *Sidalcea oregana* | 20,704  (0.9607) | 20,704  (1.0) | 35,343  (0.7779) | 35,343  (0.8244) |
|  |  | *Potentilla gracilis* | 439  (0.0204) | 0 | 4,402  (0.0969) | 4,402  (0.1027) |
|  |  | *Thermopsis montana* | 82  (0.0038) | 0 | 109  (0.0024) | 0 |
|  |  | *Vicia villosa* | 0 | 0 | 20  (0.0004) | 0 |
|  |  | Additional taxa | 279  (0.0129) | 0 | 5,312  (0.1169) | 3,128  (0.0730) |
|  | 3 | *Onopordum acanthium* | 9  (0.0005) | 0 | 63  (0.0012) | 0 |
|  |  | *Sidalcea oregana* | 18,038  (0.9683) | 18,038  (1.0) | 42,490  (0.7968) | 42,490  (0.8592) |
|  |  | *Potentilla gracilis* | 244  (0.0131) | 0 | 3,614  (0.0678) | 3,614  (0.0731) |
|  |  | *Thermopsis montana* | 57  (0.0031) | 0 | 122  (0.0023) | 0 |
|  |  | *Vicia villosa* | 5  (0.0003) | 0 | 66  (0.0012) | 0 |
|  |  | Additional taxa | 275  (0.0148) | 0 | 6,970  (0.1307) | 3,351  (0.0678) |
| *Potentilla gracilis* | 1 | *Onopordum acanthium* | 12  (0.0004) | 0 | 0 | 0 |
|  |  | *Sidalcea oregana* | 18  (0.0006) | 0 | 0 | 0 |
|  |  | *Potentilla gracilis* | 29,024  (0.9534) | 29,024  (0.9628) | 65,692  (0.9802) | 65,692  (1.0) |
|  |  | *Thermopsis montana* | 25  (0.0008) | 0 | 26  (0.0004) | 0 |
|  |  | *Vicia villosa* | 0 | 0 | 11  (0.0002) | 0 |
|  |  | Additional taxa | 1,365  (0.0448) | 1,122  (0.0372) | 1,289  (0.0192) | 0 |
|  | 2 | *Onopordum acanthium* | 10  (0.0003) | 0 | 0 | 0 |
|  |  | *Sidalcea oregana* | 0 | 0 | 16  (0.0002) | 0 |
|  |  | *Potentilla gracilis* | 30,238  (0.9494) | 30,238  (0.9606) | 64,157  (0.9845) | 64,157  (1.0) |
|  |  | *Thermopsis montana* | 33  (0.001) | 0 | 22  (0.0003) | 0 |
|  |  | *Vicia villosa* | 4  (0.0001) | 0 | 21  (0.0003) | 0 |
|  |  | Additional taxa | 1,563  (0.0491) | 1,240  (0.0394) | 949  (0.0146) | 0 |
|  | 3 | *Onopordum acanthium* | 15  (0.0003) | 0 | 0 | 0 |
|  |  | *Sidalcea oregana* | 0 | 0 | 0 | 0 |
|  |  | *Potentilla gracilis* | 42,865  (0.9477) | 42,865  (0.9583) | 50,199  (0.9828) | 50,199  (1.0) |
|  |  | *Thermopsis montana* | 21  (0.0005) | 0 | 27  (0.0005) | 0 |
|  |  | *Vicia villosa* | 0 | 0 | 0 | 0 |
|  |  | Additional taxa | 2,331  (0.0515) | 1,864  (0.0417) | 852  (0.0167) | 0 |
| *Thermopsis montana* | 1 | *Onopordum acanthium* | 10  (0.0002) | 0 | 0 | 0 |
|  |  | *Sidalcea oregana* | 0 | 0 | 0 | 0 |
|  |  | *Potentilla gracilis* | 0 | 0 | 0 | 0 |
|  |  | *Thermopsis montana* | 40,523  (0.9989) | 40,523  (1.0) | 76,528  (0.9850) | 76,528  (1.0) |
|  |  | *Vicia villosa* | 0 | 0 | 50  (0.0006) | 0 |
|  |  | Additional taxa | 35  (0.0009) | 0 | 1,118  (0.0144) | 0 |
|  | 2 | *Onopordum acanthium* | 25  (0.0007) | 0 | 40  (0.0005) | 0 |
|  |  | *Sidalcea oregana* | 10  (0.0003) | 0 | 0 | 0 |
|  |  | *Potentilla gracilis* | 0 | 0 | 0 | 0 |
|  |  | *Thermopsis montana* | 33,371  (0.999) | 33,371  (1.0) | 71,359  (0.9431) | 71,359  (0.9533) |
|  |  | *Vicia villosa* | 0 | 0 | 0 | 0 |
|  |  | Additional taxa | 0 | 0 | 4,263  (0.0563) | 3,498  (0.0467) |
|  | 3 | *Onopordum acanthium* | 13  (0.0005) | 0 | 46  (0.0006) | 0 |
|  |  | *Sidalcea oregana* | 0 | 0 | 0 | 0 |
|  |  | *Potentilla gracilis* | 0 | 0 | 40  (0.0005) | 0 |
|  |  | *Thermopsis montana* | 27,679  (0.9989) | 27,679  (1.0) | 71,274  (0.9737) | 71,274  (0.9809) |
|  |  | *Vicia villosa* | 0 | 0 | 0 | 0 |
|  |  | Additional taxa | 18  (0.0006) | 0 | 1,838  (0.0251) | 1,386  (0.0191) |
| *Vicia villosa* | 1 | *Onopordum acanthium* | 6  (0.0002) | 0 | 44  (0.0006) | 0 |
|  |  | *Sidalcea oregana* | 0 | 0 | 0 | 0 |
|  |  | *Potentilla gracilis* | 0 | 0 | 23  (0.0003) | 0 |
|  |  | *Thermopsis montana* | 42  (0.0015) | 0 | 28  (0.0004) | 0 |
|  |  | *Vicia villosa* | 27,603  (0.9979) | 27,603  (1.0) | 70,331  (0.9983) | 70,331  (1.0) |
|  |  | Additional taxa | 10  (0.0004) | 0 | 25  (0.0004) | 0 |
|  | 2 | *Onopordum acanthium* | 12  (0.0003) | 0 | 52  (0.0007) | 0 |
|  |  | *Sidalcea oregana* | 10  (0.0003) | 0 | 0 | 0 |
|  |  | *Potentilla gracilis* | 0 | 0 | 7  (0.0001) | 0 |
|  |  | *Thermopsis montana* | 28  (0.0007) | 0 | 25  (0.0003) | 0 |
|  |  | *Vicia villosa* | 38,127  (0.9986) | 38,127  (1.0) | 73,810  (0.9983) | 73,810  (1.0) |
|  |  | Additional taxa | 5  (0.0001) | 0 | 38  (0.0005) | 0 |
|  | 3 | *Onopordum acanthium* | 14  (0.0004) | 0 | 28  (0.0003) | 0 |
|  |  | *Sidalcea oregana* | 0 | 0 | 0 | 0 |
|  |  | *Potentilla gracilis* | 23  (0.0007) | 0 | 15  (0.0002) | 0 |
|  |  | *Thermopsis montana* | 12  (0.0004) | 0 | 60  (0.0007) | 0 |
|  |  | *Vicia villosa* | 31,610  (0.9982) | 31,610  (1.0) | 82,421  (0.9983) | 82,421  (1.0) |
|  |  | Additional taxa | 8  (0.0003) | 0 | 35  (0.0004) | 0 |

See S4 Table for a list of additional taxa identified in pollen samples. Additional taxa are defined as plant taxa detected in the samples that were not used to create the laboratory-prepared pollen mixtures. See S9 Table for taxonomic assignments using ITS2 and *rbcL* metabarcoding.
