# Supplementary material for: Investigating the use of pollen DNA metabarcoding to quantify bee foraging and effects of threshold selection
Source: PLoS One. 2023 Apr 18;18(4):e0282715. doi: 10.1371/journal.pone.0282715 (PMC10112814; doi:10.1371/journal.pone.0282715)
Supplement: S3 Table — Shaded boxes represent instances in which the conservative threshold failed to detect species in mixture, resulting in false negatives. See table in S4 Table for a list of additional taxa identified in mixture samples. Additional taxa are defined as plant taxa detected in the samples that were not used to create the laboratory-prepared pollen mixtures. See table in S9 Table for taxonomic assignments using ITS2 and rbcL metabarcoding. (DOCX) [file pone.0282715.s005.docx]

**S3 Table**. **Proportion by mass and number (proportion) of ITS2 and *rbcL* sequencing reads for each plant species (and their taxonomic assignments using metabarcoding) in laboratory-prepared mixtures (three replicates/mixture) using liberal and conservative thresholds.**

| **Mixture** | **Replicate** | **Species Composition** | **Proportion by mass** | **Number (proportion) of ITS2 sequence reads** | | **Number (proportion) of rbcL sequence reads** | |
| --- | --- | --- | --- | --- | --- | --- | --- |
|  |  |  |  | Liberal threshold | Conservative threshold | Liberal threshold | Conservative threshold |
| 1 | 1 | *Onopordum acanthium* | 0.47 | 25,103  (0.58) | 25,103  (0.59) | 22,667  (0.33) | 22,667  (0.34) |
|  |  | *Sidalcea oregana* | 0.28 | 2,881  (0.07) | 2,881  (0.07) | 3,673  (0.05) | 3,673  (0.06) |
|  |  | *Potentilla gracilis* | 0.25 | 14,544  (0.33) | 14,544  (0.34) | 40,190  (0.58) | 40,190  (0.60) |
|  |  | *Thermopsis montana* | 0 | 11  (0.0003) | 0 | 46  (0.001) | 0 |
|  |  | *Vicia villosa* | 0 | 0 | 0 | 0 | 0 |
|  |  | Additional taxa | 0 | 1,067  (0.02) | 0 | 2,331  (0.03) | 0 |
|  | 2 | *Onopordum acanthium* | 0.47 | 20,032  (0.57) | 20,032  (0.58) | 24,737  (0.38) | 24,737  (0.39) |
|  |  | *Sidalcea oregana* | 0.28 | 2,852  (0.08) | 2,852  (0.08) | 3,211  (0.05) | 3,211  (0.05) |
|  |  | *Potentilla gracilis* | 0.25 | 11,422  (0.32) | 11,422  (0.33) | 35,085  (0.53) | 35,085  (0.56) |
|  |  | *Thermopsis montana* | 0 | 20  (0.001) | 0 | 21  (0.0003) | 0 |
|  |  | *Vicia villosa* | 0 | 0 | 0 | 0 | 0 |
|  |  | Additional taxa | 0 | 902  (0.03) | 0 | 2,755  (0.04) | 0 |
|  | 3 | *Onopordum acanthium* | 0.47 | 31,110  (0.54) | 31,110  (0.55) | 22,431  (0.41) | 22,431  (0.42) |
|  |  | *Sidalcea oregana* | 0.28 | 6,925  (0.12) | 6,925  (0.12) | 4,065  (0.07) | 4,065  (0.08) |
|  |  | *Potentilla gracilis* | 0.25 | 18,412  (0.32) | 18,412  (0.33) | 25,031  (0.46) | 25,031  (0.47) |
|  |  | *Thermopsis montana* | 0 | 53  (0.001) | 0 | 6  (0.0001) | 0 |
|  |  | *Vicia villosa* | 0 | 0 | 0 | 18  (0.0003) | 0 |
|  |  | Additional taxa | 0 | 1,550  (0.03) | 0 | 2,946  (0.05) | 1,494  (0.03) |
| 2 | 1 | *Onopordum acanthium* | 0.40 | 3,495  (0.07) | 3,495  (0.07) | 4,322  (0.09) | 4,322  (0.09) |
|  |  | *Sidalcea oregana* | 0.20 | 254  (0.01) | 0 | 297  (0.01) | 0 |
|  |  | *Potentilla gracilis* | 0.20 | 2,712  (0.05) | 2,712  (0.05) | 7,530  (0.16) | 7,530  (0.17) |
|  |  | *Thermopsis montana* | 0.20 | 43,596  (0.87) | 43,596  (0.88) | 33,734  (0.71) | 33,734  (0.74) |
|  |  | *Vicia villosa* | 0 | 0 | 0 | 15  (0.0003) | 0 |
|  |  | Additional taxa | 0 | 196  (0.004) | 0 | 1,856  (0.04) | 0 |
|  | 2 | *Onopordum acanthium* | 0.40 | 4,660  (0.09) | 4,660  (0.09) | 4,440  (0.09) | 4,440  (0.09) |
|  |  | *Sidalcea oregana* | 0.20 | 351  (0.01) | 0 | 414  (0.01) | 0 |
|  |  | *Potentilla gracilis* | 0.20 | 2,813  (0.05) | 2,813  (0.05) | 7,810  (0.15) | 7,810  (0.16) |
|  |  | *Thermopsis montana* | 0.20 | 43,746  (0.84) | 43,746  (0.85) | 37,356  (0.72) | 37,356  (0.75) |
|  |  | *Vicia villosa* | 0 | 0 | 0 | 0 | 0 |
|  |  | Additional taxa | 0 | 226  (0.004) | 0 | 1,872  (0.04) | 0 |
|  | 3 | *Onopordum acanthium* | 0.40 | 3,600  (0.07) | 3,600  (0.07) | 5,041  (0.10) | 5,041  (0.10) |
|  |  | *Sidalcea oregana* | 0.20 | 308  (0.01) | 0 | 378  (0.01) | 0 |
|  |  | *Potentilla gracilis* | 0.20 | 2,615  (0.05) | 2,615  (0.05) | 8,292  (0.16) | 8,292  (0.17) |
|  |  | *Thermopsis montana* | 0.20 | 43,815  (0.87) | 43,815  (0.88) | 35,618  (0.70) | 35,618  (0.73) |
|  |  | *Vicia villosa* | 0 | 0 | 0 | 37  (0.001) | 0 |
|  |  | Additional taxa | 0 | 217  (0.004) | 0 | 1,804  (0.04) | 0 |
| 3 | 1 | *Onopordum acanthium* | 0.20 | 2,584  (0.06) | 2,584  (0.06) | 3,590  (0.08) | 3,590  (0.09) |
|  |  | *Sidalcea oregana* | 0.20 | 249  (0.01) | 0 | 325  (0.01) | 0 |
|  |  | *Potentilla gracilis* | 0.20 | 1,265  (0.03) | 1,265  (0.03) | 2,938  (0.07) | 2,938  (0.07) |
|  |  | *Thermopsis montana* | 0.20 | 33,638  (0.78) | 33,638  (0.79) | 21,714  (0.50) | 21,714  (0.51) |
|  |  | *Vicia villosa* | 0.20 | 5,145  (0.12) | 5,145  (0.12) | 14,006  (0.32) | 14,006  (0.33) |
|  |  | Additional taxa | 0 | 133  (0.003) | 0 | 1,037  (0.02) | 0 |
|  | 2 | *Onopordum acanthium* | 0.20 | 1,560  (0.03) | 1,560  (0.03) | 3,834  (0.06) | 3,834  (0.06) |
|  |  | *Sidalcea oregana* | 0.20 | 128  (0.003) | 0 | 242  (0.004) | 0 |
|  |  | *Potentilla* gracilis | 0.20 | 1,009  (0.02) | 1,009  (0.02) | 3,713  (0.05) | 3,713  (0.05) |
|  |  | *Thermopsis montana* | 0.20 | 40,515  (0.87) | 40,515  (0.88) | 44,688  (0.64) | 44,688  (0.66) |
|  |  | *Vicia villosa* | 0.20 | 3,075  (0.07) | 3,075  (0.07) | 15,696  (0.23) | 15,696  (0.23) |
|  |  | Additional taxa | 0 | 122  (0.003) | 0 | 1,586  (0.03) | 0 |
|  | 3 | *Onopordum acanthium* | 0.20 | 1,509  (0.04) | 1,509  (0.04) | 3,836  (0.08) | 3,836  (0.09) |
|  |  | *Sidalcea oregana* | 0.20 | 258  (0.01) | 0 | 340  (0.01) | 0 |
|  |  | *Potentilla gracilis* | 0.20 | 900  (0.02) | 0 | 3,362  (0.07) | 3,362  (0.07) |
|  |  | *Thermopsis montana* | 0.20 | 32,875  (0.87) | 32,875  (0.90) | 27,099  (0.58) | 27,099  (0.60) |
|  |  | *Vicia villosa* | 0.20 | 2,248  (0.06) | 2,248  (0.06) | 10,733  (0.23) | 10,733  (0.24) |
|  |  | Additional taxa | 0 | 85  (0.002) | 0 | 1,574  (0.03) | 0 |
| 4 | 1 | *Onopordum acanthium* | 0.60 | 10,050  (0.21) | 10,050  (0.21) | 13,430  (0.30) | 13,430  (0.31) |
|  |  | *Sidalcea oregana* | 0.10 | 60  (0.001) | 0 | 26  (0.001) | 0 |
|  |  | *Potentilla gracilis* | 0.10 | 2,371  (0.05) | 2,371  (0.05) | 3,728  (0.08) | 3,728  (0.09) |
|  |  | *Thermopsis montana* | 0.10 | 28,980  (0.61) | 28,980  (0.61) | 11,675  (0.26) | 11,675  (0.27) |
|  |  | *Vicia villosa* | 0.10 | 5,753  (0.12) | 5,753  (0.12) | 14,683  (0.33) | 14,683  (0.34) |
|  |  | Additional taxa | 0 | 184  (0.004) | 0 | 1,012  (0.02) | 0 |
|  | 2 | *Onopordum acanthium* | 0.60 | 9,290  (0.26) | 9,290  (0.26) | 15,032  (0.37) | 15,032  (0.38) |
|  |  | *Sidalcea oregana* | 0.10 | 58  (0.002) | 0 | 62  (0.002) | 0 |
|  |  | *Potentilla gracilis* | 0.10 | 1,516  (0.04) | 1,516  (0.04) | 3,439  (0.08) | 3,439  (0.09) |
|  |  | *Thermopsis montana* | 0.10 | 20,672  (0.58) | 20,672  (0.59) | 11,055  (0.27) | 11,055  (0.28) |
|  |  | *Vicia villosa* | 0.10 | 3,840  (0.11) | 3,840  (0.11) | 10,421  (0.25) | 10,421  (0.26) |
|  |  | Additional taxa | 0 | 108  (0.003) | 0 | 894  (0.02) | 0 |
|  | 3 | *Onopordum acanthium* | 0.60 | 10,752  (0.31) | 10,752  (0.31) | 11,777  (0.40) | 11,777  (0.40) |
|  |  | *Sidalcea oregana* | 0.10 | 63  (0.002) | 0 | 54  (0.002) | 0 |
|  |  | *Potentilla gracilis* | 0.10 | 1,198  (0.03) | 1,198  (0.04) | 2,136  (0.07) | 2,136  (0.07) |
|  |  | *Thermopsis montana* | 0.10 | 19,458  (0.57) | 19,458  (0.57) | 8,289  (0.27) | 8,289  (0.28) |
|  |  | *Vicia villosa* | 0.10 | 2,839  (0.08) | 2,839  (0.08) | 7,436  (0.25) | 7,436  (0.25) |
|  |  | Additional taxa | 0 | 127  (0.004) | 0 | 573  (0.02) | 0 |
| 5 | 1 | *Onopordum acanthium* | 0.81 | 21,199  (0.68) | 21,199  (0.71) | 41,450  (0.75) | 41,450  (0.77) |
|  |  | *Sidalcea oregana* | 0.10 | 85  (0.003) | 0 | 139  (0.003) | 0 |
|  |  | *Potentilla gracilis* | 0.03 | 493  (0.02) | 0 | 2,253  (0.04) | 2,253  (0.04) |
|  |  | *Thermopsis montana* | 0.03 | 8,825  (0.28) | 8,825  (0.29) | 7,146  (0.13) | 7,146  (0.13) |
|  |  | *Vicia villosa* | 0.03 | 707  (0.02) | 0 | 3,223  (0.06) | 3,223  (0.06) |
|  |  | Additional taxa | 0 | 97  (0.003) | 0 | 811  (0.01) | 0 |
|  | 2 | *Onopordum acanthium* | 0.81 | 22,751  (0.69) | 22,751  (0.72) | 45,408  (0.79) | 45,408  (0.80) |
|  |  | *Sidalcea oregana* | 0.10 | 226  (0.01) | 0 | 191  (0.003) | 0 |
|  |  | *Potentilla gracilis* | 0.03 | 376  (0.01) | 0 | 1,381  (0.02) | 1,381  (0.02) |
|  |  | *Thermopsis montana* | 0.03 | 8,667  (0.26) | 8,667  (0.28) | 6,330  (0.11) | 6,330  (0.11) |
|  |  | *Vicia villosa* | 0.03 | 73  (0.02) | 0 | 3,448  (0.06) | 3,448  (0.06) |
|  |  | Additional taxa | 0 | 87  (0.002) | 0 | 550  (0.01) | 0 |
|  | 3 | *Onopordum acanthium* | 0.81 | 13,383  (0.53) | 13,383  (0.54) | 36,062  (0.71) | 36,062  (0.75) |
|  |  | *Sidalcea oregana* | 0.10 | 190  (0.08) | 0 | 441  (0.01) | 0 |
|  |  | *Potentilla gracilis* | 0.03 | 132  (0.01) | 0 | 961  (0.02) | 0 |
|  |  | *Thermopsis montana* | 0.03 | 11,214  (0.44) | 11,214  (0.46) | 10,800  (0.21) | 10,800  (0.22) |
|  |  | *Vicia villosa* | 0.03 | 247  (0.01) | 0 | 1,491  (0.03) | 1,491  (0.03) |
|  |  | Additional taxa | 0 | 53  (0.002) | 0 | 826  (0.02) | 0 |

Shaded boxes represent instances in which the conservative threshold failed to detect species in mixture, resulting in false negatives. See S4 Table for a list of additional taxa identified in mixture samples. Additional taxa are defined as plant taxa detected in the samples that were not used to create the laboratory-prepared pollen mixtures. See S9 Table for taxonomic assignments using ITS2 and *rbcL* metabarcoding.
