# Supplementary material for: Investigating the use of pollen DNA metabarcoding to quantify bee foraging and effects of threshold selection
Source: PLoS One. 2023 Apr 18;18(4):e0282715. doi: 10.1371/journal.pone.0282715 (PMC10112814; doi:10.1371/journal.pone.0282715)
Supplement: S4 Table — Laboratory-prepared mixtures were created from the same pollen stocks used to create the single-species samples. “M” denotes a plant taxon that was detected in a mixture sample, “S” denotes a plant taxon that was detected in a single-species sample, and “MS” denotes a plant taxon that was detected in both a mixture sample and a single-species sample. (DOCX) [file pone.0282715.s006.docx]

**S4 Table.** **Plant taxa that were not used to create the laboratory-prepared mixtures but were detected in mixtures and single-species samples.**

| Plant Taxa | ITS2 | | *rbcL* | |
| --- | --- | --- | --- | --- |
|  | **Liberal** | **Conservative** | **Liberal** | **Conservative** |
| *Achillea millefolium* | MS |  | MS |  |
| *Allium schoenoprasum* | MS |  |  |  |
| *Asparagus officinalis* |  |  | MS |  |
| *Erigeron* sp*.* |  |  | MS |  |
| *Eriogonum flavum* |  |  | S |  |
| *Holodiscus discolor* |  |  | MS |  |
| *Hypericum perforatum* | MS |  | MS | MS |
| *Iris pseudacorus* |  |  | MS |  |
| *Lupinus* sp. |  |  | MS |  |
| *Lupinus arbustus* | MS |  |  |  |
| *Mentha canadensis* |  |  | MS |  |
| *Penstemon* sp. | M |  | MS |  |
| *Pinus contorta* |  |  | MS | S |
| *Pinus ponderosa* |  |  | MS |  |
| *Populus* sp. |  |  | MS |  |
| *Potentilla norvegica* | MS |  |  |  |
| *Potentilla recta* | MS | S |  |  |
| *Ranunculus* sp. |  |  | M |  |
| *Sambucus racemosa* |  |  | MS |  |
| *Solanum* sp. |  |  | MS | S |
| *Solidago lepida* | MS |  |  |  |
| *Spirea* sp. |  |  | MS |  |
| *Symphoricarpos albus* |  |  | MS |  |
| *Symphyotrichum* sp. | MS |  |  |  |
| *Trifolium hybridum* | MS |  |  |  |
| *Trifolium repens* |  |  | MS | S |
| *Typha angustifolia* |  |  | MS |  |

Laboratory-prepared mixtures were created from the same pollen stocks used to create the single-species samples. “M” denotes a plant taxon that was detected in a mixture sample, “S” denotes a plant taxon that was detected in a single-species sample, and “MS” denotes a plant taxon that was detected in both a mixture sample and a single-species sample.
