# Supplementary material for: Investigating the use of pollen DNA metabarcoding to quantify bee foraging and effects of threshold selection
Source: PLoS One. 2023 Apr 18;18(4):e0282715. doi: 10.1371/journal.pone.0282715 (PMC10112814; doi:10.1371/journal.pone.0282715)
Supplement: S5 Table — (DOCX) [file pone.0282715.s007.docx]

**S5 Table. Complete list of bee species included in Fig 4.**

| **Network ID** | **Bee Species** |
| --- | --- |
| 1 | *Bombus bifarius* |
| 2 | *Bombus californicus* |
| 3 | *Halictus farinosus* |
| 4 | *Halictus ligatus* |
| 5 | *Halictus tripartitus* |
| 6 | *Melissodes microstictus* |
