# Supplementary material for: Investigating the use of pollen DNA metabarcoding to quantify bee foraging and effects of threshold selection
Source: PLoS One. 2023 Apr 18;18(4):e0282715. doi: 10.1371/journal.pone.0282715 (PMC10112814; doi:10.1371/journal.pone.0282715)
Supplement: S6 Table — (DOCX) [file pone.0282715.s008.docx]

**S6 Table.** **Complete list of plant taxa included in Fig 4A.**

| **Network ID** | **Plant Taxa** |
| --- | --- |
| X1 | Apiaceae |
| X2 | *Heracleum maximum* |
| X2.1 | *Lomatium gormanii* |
| X3 | *Allium* sp. |
| X4 | *Triteleia grandiflora* |
| X5 | Asteraceae |
| X6 | *Achillea millefolium* |
| X7 | *Antennaria* sp. |
| X8 | *Arctium minus* |
| X9 | *Artemisia* sp. |
| X10 | *Artemisia ludoviciana* |
| X11 | *Erigeron* sp. |
| X12 | *Erigeron corymbosus* |
| X13 | *Hieracium scouleri* |
| X14 | *Packera* sp. |
| X15 | *Pyrrocoma carthamoides* |
| X16 | *Senecio integerrimus* |
| X17 | *Solidago canadensis* |
| X18 | *Solidago missouriensis* |
| X19 | *Symphyotrichum* sp. |
| X20 | *Symphyotrichum spathulatum* |
| X21 | *Dianthus armeria* |
| X22 | *Polygonum douglasii* |
| X23 | *Sambucus nigra* |
| X24 | *Symphoricarpos albus* |
| X25 | *Lupinus* sp. |
| X26 | *Lupinus leucophyllus* |
| X27 | *Medicago sativa* |
| X28 | *Thermopsis montana* |
| X29 | *Trifolium* sp. |
| X30 | *Trifolium repens* |
| X31 | *Vicia cracca* |
| X32 | *Gentianella tenella* |
| X33 | *Monardella odoratissima* |
| X34 | *Calochortus macrocarpus* |
| X35 | *Hypericum* sp. |
| X36 | *Hypericum perforatum* |
| X37 | *Hypericum scouleri* |
| X38 | *Sidalcea oregana* |
| X39 | *Ranunculaceae* |
| X40 | *Holodiscus discolor* |
| X41 | *Potentilla gracilis* |
| X42 | *Potentilla recta* |
| X43 | *Sanguisorba canadensis* |
| X44 | *Spiraea betulifolia* |
