# Supplementary material for: Investigating the use of pollen DNA metabarcoding to quantify bee foraging and effects of threshold selection
Source: PLoS One. 2023 Apr 18;18(4):e0282715. doi: 10.1371/journal.pone.0282715 (PMC10112814; doi:10.1371/journal.pone.0282715)
Supplement: S7 Table — (DOCX) [file pone.0282715.s009.docx]

**S7 Table.** **Complete list of plant taxa included in Fig 4B.**

| **Network ID** | **Plant Taxa** |
| --- | --- |
| 1 | Apiaceae |
| 2 | Asteraceae |
| 3 | *Achillea millefolium* |
| 4 | *Packera* sp. |
| 5 | *Pyrrocoma carthamoides* |
| 6 | *Senecio integerrimus* |
| 7 | *Solidago canadensis* |
| 8 | *Symphyotrichum* sp. |
| 9 | *Symphyotrichum spathulatum* |
| 10 | *Dianthus armeria* |
| 11 | *Symphoricarpos albus* |
| 12 | *Lupinus leucophyllus* |
| 13 | *Trifolium repens* |
| 14 | *Hypericum perforatum* |
| 15 | *Sanguisorba canadensis* |
