# Supplementary material for: Investigating the use of pollen DNA metabarcoding to quantify bee foraging and effects of threshold selection
Source: PLoS One. 2023 Apr 18;18(4):e0282715. doi: 10.1371/journal.pone.0282715 (PMC10112814; doi:10.1371/journal.pone.0282715)
Supplement: S8 Table — (DOCX) [file pone.0282715.s010.docx]

**S8 Table. Mass and DNA yield for each pollen sample.**

| **Sample** | **Replicate** | **Mass (mg)** | **DNA yield (ng/μL)** |
| --- | --- | --- | --- |
| Mixture 1 | 1 | 2.5 | 114.1 |
| Mixture 1 | 2 | 2.5 | 95.8 |
| Mixture 1 | 3 | 2.4 | 31.7 |
| Mixture 2 | 1 | 2.4 | 193.3 |
| Mixture 2 | 2 | 2.6 | 170.7 |
| Mixture 2 | 3 | 2.7 | 82.3 |
| Mixture 3 | 1 | 2.6 | 199.8 |
| Mixture 3 | 2 | 2.5 | 311.3 |
| Mixture 3 | 3 | 2.4 | 196.1 |
| Mixture 4 | 1 | 2.5 | 145 |
| Mixture 4 | 2 | 2.6 | 123.7 |
| Mixture 4 | 3 | 2.6 | 182.9 |
| Mixture 5 | 1 | 2.5 | 107.1 |
| Mixture 5 | 2 | 2.4 | 93.6 |
| Mixture 5 | 3 | 2.7 | 90.4 |
| *Onopordum acanthium* | 1 | 1.2 | 29.2 |
| *Onopordum acanthium* | 2 | 1.0 | 19.9 |
| *Onopordum acanthium* | 3 | 1.2 | 15.6 |
| *Sidalcea oregana* | 1 | 1.5 | 30.2 |
| *Sidalcea oregana* | 2 | 1.6 | 46.3 |
| *Sidalcea oregana* | 3 | 1.6 | 30.7 |
| *Potentilla gracilis* | 1 | 1.3 | 109.8 |
| *Potentilla gracilis* | 2 | 1.2 | 85.4 |
| *Potentilla gracilis* | 3 | 1.5 | 89.6 |
| *Thermopsis montana* | 1 | 1.2 | 326.9 |
| *Thermopsis montana* | 2 | 1.0 | 207.6 |
| *Thermopsis montana* | 3 | 1.5 | 276.3 |
| *Vicia villosa* | 1 | 1.5 | 217.3 |
| *Vicia villosa* | 2 | 1.4 | 394.4 |
| *Vicia villosa* | 3 | 1.5 | 183.9 |
| Bee Pollen 1 | NA | 5.7 | 241.2 |
| Bee Pollen 2 | NA | 2.9 | 226.5 |
| Bee Pollen 3 | NA | 16.5 | 621.9 |
| Bee Pollen 4 | NA | 3.8 | 102.2 |
| Bee Pollen 5 | NA | 10.4 | 558.4 |
| Bee Pollen 6 | NA | <1.0 | 3.5 |
| Bee Pollen 7 | NA | <1.0 | 7.2 |
| Bee Pollen 8 | NA | <1.0 | 11.5 |
| Bee Pollen 9 | NA | <1.0 | 29.6 |
| Bee Pollen 10 | NA | <1.0 | 3.4 |
| Bee Pollen 11 | NA | <1.0 | 4.0 |
| Bee Pollen 12 | NA | <1.0 | 8.3 |
| Bee Pollen 13 | NA | <1.0 | 1.2 |
