# Supplementary material for: Investigating the use of pollen DNA metabarcoding to quantify bee foraging and effects of threshold selection
Source: PLoS One. 2023 Apr 18;18(4):e0282715. doi: 10.1371/journal.pone.0282715 (PMC10112814; doi:10.1371/journal.pone.0282715)
Supplement: S9 Table — (DOCX) [file pone.0282715.s011.docx]

**S9 Table. Plant taxonomic assignments from ITS2 and *rbcL* metabarcoding.**

| **Plant Species in Mixtures** | **ITS2 Assignment** | ***rbcL* Assignment** |
| --- | --- | --- |
| *Onopordum acanthium* | *Onopordum acanthium* | *Hieracium* sp. |
| *Sidalcea oregana* | *Sidalcea oregana* | *Malva neglecta* |
| *Potentilla gracilis* | *Potentilla* sp. | *Potentilla* sp. |
| *Thermopsis montana* | *Thermopsis rhombifolia* | *Thermopsis* sp. |
| *Vicia villosa* | *Vicia villosa* | *Vicia villosa* |
