# Supplementary material for: Investigating the use of pollen DNA metabarcoding to quantify bee foraging and effects of threshold selection
Source: PLoS One. 2023 Apr 18;18(4):e0282715. doi: 10.1371/journal.pone.0282715 (PMC10112814; doi:10.1371/journal.pone.0282715)
Supplement: S10 Table — Extraction, PCR and Plate refers to negative controls that were collected during DNA isolation, PCR, and plating respectively. (DOCX) [file pone.0282715.s012.docx]

**S10 Table. Plant species and corresponding number of sequencing reads found in each of the negative controls.**

|  | **Extraction 1** | | **Extraction 2** | | **PCR 1** | | **PCR 2** | | **Plate 1** | |
| --- | --- | --- | --- | --- | --- | --- | --- | --- | --- | --- |
|  | **ITS2** | ***rbcL*** | **ITS2** | ***rbcL*** | **ITS2** | ***rbcL*** | **ITS2** | ***rbcL*** | **ITS2** | ***rbcL*** |
| ***Achillea millefolium*** | 0 | 0 | 0 | 0 | 0 | 0 | 4 | 0 | 0 | 0 |
| ***Centaurea diffusa*** | 0 | 0 | 94 | 0 | 0 | 0 | 0 | 0 | 0 | 0 |
| ***Dieteria canescens*** | 0 | 0 | 83 | 0 | 0 | 0 | 0 | 0 | 0 | 0 |
| ***Eriophyllum lanatum*** | 147 | 0 | 0 | 0 | 0 | 0 | 0 | 0 | 0 | 0 |
| ***Onopordum acanthium*** | 5 | 0 | 0 | 0 | 0 | 0 | 0 | 0 | 9 | 0 |
| ***Solidago lepida*** | 11 | 0 | 10 | 0 | 0 | 0 | 0 | 0 | 0 | 0 |
| ***Sinapis arvensis*** | 444 | 0 | 0 | 0 | 0 | 0 | 0 | 0 | 0 | 0 |
| ***Lupinus arbustus*** | 330 | 0 | 0 | 0 | 39 | 0 | 7 | 0 | 9 | 0 |
| ***Thermopsis rhombifolia*** | 17 | 0 | 0 | 0 | 307 | 0 | 0 | 0 | 38 | 0 |
| ***Vicia villosa*** | 0 | 0 | 0 | 0 | 0 | 29 | 0 | 0 | 4 | 0 |
| ***Frasera fastigiata*** | 0 | 0 | 0 | 0 | 0 | 0 | 118 | 0 | 0 | 0 |
| ***Gentianella tenella*** | 0 | 0 | 0 | 0 | 0 | 0 | 0 | 0 | 4 | 0 |
| ***Castilleja cusickii*** | 65 | 0 | 0 | 0 | 0 | 0 | 0 | 0 | 0 | 0 |
| ***Penstemon* sp.** | 0 | 0 | 0 | 0 | 0 | 0 | 120 | 0 | 0 | 0 |
| ***Sidalcea oregana*** | 0 | 0 | 0 | 0 | 9 | 0 | 0 | 0 | 0 | 0 |
| ***Potentilla* sp.** | 0 | 0 | 0 | 0 | 0 | 13 | 12 | 0 | 0 | 0 |
| ***Erigeron* sp.** | 0 | 0 | 0 | 0 | 0 | 883 | 0 | 0 | 0 | 0 |
| ***Thermopsis* sp.** | 0 | 0 | 0 | 0 | 0 | 43 | 0 | 0 | 0 | 0 |
| ***Hypericum perforatum*** | 0 | 0 | 0 | 0 | 0 | 21 | 0 | 0 | 0 | 0 |
| ***Ranunculus* sp.** | 0 | 0 | 0 | 0 | 0 | 1309 | 0 | 0 | 0 | 0 |

Extraction, PCR and Plate refers to negative controls that were collected during DNA isolation, PCR, and plating respectively.
